# Supplementary material for: Unraveling activation-related rearrangements and intrinsic divergence from ligand-induced conformational changes of the dopamine D3 and D2 receptors
Source: bioRxiv. 2023 Nov 14:2023.11.11.566699. Preprint. [Version 1] doi: 10.1101/2023.11.11.566699 (PMC10680602; doi:10.1101/2023.11.11.566699)
Supplement: 1 [file NIHPP2023.11.11.566699V1-supplement-1.pdf]

bioRxiv preprint doi: https://doi.org/10.1101/2023.11.11.566699; this version posted November 14, 2023. The copyright holder for this preprint (which was not certified by peer review) is the author/funder. This article is a US Government work. It is not subject to copyright under 17 USC 105 and is also made available for use under a CC0 license.

Supplemental Materials

Table S1. The experimentally determined D2R and D3R structures used in this study.

condition	PDB ID	publication year	resolution (Å)	bound ligand	reference
**D2R inactive**	7DFP	2020	3.1	spiperone	1
	6LUQ	2020	3.1	haloperidol	2
	6CM4	2018	2.9	risperidone	3
**D2R active**	7JVR	2021	2.8	bromocriptine	4
**D3R inactive**	3PBL	2010	2.9	eticlopride	5
**D3R active**	7CMU	2021	3.0	pramipexole	6
	7CMV	2021	2.7	PD128907	6

Note that we chose to use the structure 7JVR because it has a higher resolution than the other D2R/G_i-bromocriptine structure (PDB 6VMS)[7].

bioRxiv preprint doi: https://doi.org/10.1101/2023.11.11.566699; this version posted November 14, 2023. The copyright holder for this preprint (which was not certified by peer review) is the author/funder. This article is a US Government work. It is not subject to copyright under 17 USC 105 and is also made available for use under a CC0 license.

Table S2. Sequence alignment of TM1m and TM1i

**TM1m-TM1i**	**TM1m TM1i**<br>1.37 1.45 1.46 1.59<br>| | | | **D3R LSYCALILA IVFGNGLVCMAVLK**<br>**D2R TLLTLLIAV IVFGNVLVCMAVSR**
**IL2-TM4i**	**IL2 TM4i**<br>| 4.39 4.49<br>| | | **D3R PVHYQHGTGQSS CRRVALMITAV**<br>**D2R PMLYN--TRYSS KRRVTVMISIV**
**TM5e**	**TM5e**<br>5.36 5.45<br>| | **D3R PDFVIYSSVV**<br>**D2R PAFVVYSSIV**
**TM6e-EL3-TM7e**	**TM6e EL3 TM7e**<br>6.49 6.60 | 7.32 7.43<br>| | | | | **D3R LPFFLTHVLNTH CQTCHVS PELYSATTWLGY**<br>**D2R LPFFITHILNIH CD-CNIP PVLYSAFTWLGY**

Conserved residues between D2R and D3R are highlighted in green, while divergent residues are colored in gray.

bioRxiv preprint doi: https://doi.org/10.1101/2023.11.11.566699; this version posted November 14, 2023. The copyright holder for this preprint (which was not certified by peer review) is the author/funder. This article is a US Government work. It is not subject to copyright under 17 USC 105 and is also made available for use under a CC0 license.

Table S3. Prokink bend angles of TM5, TM6 and TM7.

source	state	condition	TM5	TM6	TM7
structure	D3R inactive	3PBL	1.3	20.9	27.2
	D2R inactive	6CM4	4.1	30.9	25.7
		6LUQ	2.9	34.1	28.0
		7DFP	0.9	32.8	27.7
	inactive difference	3PBL – 6CM4	-2.8	-10.0	1.5
		3PBL – 6LUQ	-1.6	-13.2	-0.8
		3PBL – 7DFP	0.4	-11.9	-0.5
	**average of inactive difference**		**-1.3**	**-11.7**	**0.1**
	D3R active	7CMV (PD128907)	2.6	19.8	21.0
		7CMU (pramipexole)	0.4	15.9	24.0
	D2R active	7JVR (bromocriptine)	4.8	34.7	15.7
	active difference	7CMV (PD128907) – 7JVR (bromocriptine)	-2.2	-14.9	5.3
		7CMU (pramipexole) – 7JVR (bromocriptine)	-4.4	-18.8	8.3
	**average of active difference**		**-3.3**	**-16.9**	**6.8**
MD	D3R active	D3R/G_i-PD128907	6.6	22.4	31.4
		D3R/G_i-pramipexole	5.9	15.6	30.2
	D2R active	D2R/G_i-bromocriptine	7.8	32.6	23.6
	active difference	D3R/G_i-PD128907 - D2R/G_i-bromocriptine	-1.2	-10.3	7.8
		D3R/G_i-pramipexole - D2R/G_i-bromocriptine	-2.0	-17.0	6.6
	**average of active difference**		**-1.6**	**-13.7**	**7.2**
	D3R active	D3R/G_i-5R-quinpirole	5.7	14.2	26.9
	D2R active	D2R/G_i-5R-quinpirole	10.0	20.0	30.7
	**D3R/G_i-5R-quinpirole – D2R/G_i-5R-quinpirole**		**-4.3**	**-5.7**	**-3.8**

The Prokink bend angle of individual structure or MD frame was first calculated with Simulaid.[8]. The inactive average is the average of 3PBL-6CM4, 3PBL-6LUQ, and 3PBL-7DFP. The active average is the average of 7CMU-7JVR, and 7CMV-7JVR. For the MD simulations, from the bend angle distributions of bootstrap ensembles for each condition, we identified the peak values of these distributions, and calculated the differences between the indicated conditions. Finally, we computed the control MD average using D3R/G_i-PD128907 - D2R/Gi-bromocriptine and D3R/G_i-pramipexole - D2R/G_i-bromocriptine.

bioRxiv preprint doi: https://doi.org/10.1101/2023.11.11.566699; this version posted November 14, 2023. The copyright holder for this preprint (which was not certified by peer review) is the author/funder. This article is a US Government work. It is not subject to copyright under 17 USC 105 and is also made available for use under a CC0 license.

Table S4. MD simulation summary.

protein complex	bound ligand	runs	length (μs)
D2R/Gi	5S-quinpirole	5	3.8
D2R/Gi	5R-quinpirole	6	10.8
D2R/Gi	bromocriptine	6	10.8
D3R/Gi	5S-quinpirole	8	11.3
D3R/Gi	5R-quinpirole	6	10.8
D3R/Gi	pramipexole	6	10.8
D3R/Gi	PD128907	6	10.8

bioRxiv preprint doi: https://doi.org/10.1101/2023.11.11.566699; this version posted November 14, 2023. The copyright holder for this preprint (which was not certified by peer review) is the author/funder. This article is a US Government work. It is not subject to copyright under 17 USC 105 and is also made available for use under a CC0 license.

Table S5. Ligand contact frequency observed with the 5S-quinpirole and 5R-quinpirole bound D3R and D2R MD simulations.

BW index	D3R				D2R			
	residue ID	5S-quinpirole	5R-quinpirole	5S-5R	residue ID	5S-quinpirole	5R-quinpirole	5S-5R
TM3.32	110	100%	100%	0%	114	98%	100%	-2%
TM3.33	111	100%	100%	0%	115	99%	99%	0%
TM3.36	114	100%	100%	0%	118	99%	100%	-1%
TM3.37	115	4%	96%	-92%	119	72%	96%	-24%
EL2.52	183	97%	94%	3%	184	81%	95%	-14%
TM5.38	188	95%	13%	82%	189	34%	6%	28%
TM5.39	189	93%	0%	93%	190	54%	6%	48%
TM5.42	192	96%	80%	16%	193	73%	76%	-3%
TM5.46	196	24%	99%	-75%	197	97%	98%	-1%
TM6.48	342	81%	100%	-19%	386	84%	100%	-16%
TM6.51	345	100%	100%	0%	389	100%	100%	0%
TM6.52	346	89%	100%	-11%	390	100%	99%	1%
TM6.55	349	100%	100%	0%	393	94%	99%	-5%
TM7.35	365	68%	83%	-15%	408	17%	38%	-21%
TM7.39	369	97%	100%	-3%	412	81%	100%	-19%
TM7.42	372	0%	99%	-99%	415	51%	99%	-48%
TM7.43	373	65%	99%	-34%	416	76%	98%	-22%

The OBS residues are highlighted in cyan. The significantly higher contact frequencies (>40%) in the 5S-quinpirole and 5R-quinpirole bound conditions are colored in blue and red, respectively.

bioRxiv preprint doi: https://doi.org/10.1101/2023.11.11.566699; this version posted November 14, 2023. The copyright holder for this preprint (which was not certified by peer review) is the author/funder. This article is a US Government work. It is not subject to copyright under 17 USC 105 and is also made available for use under a CC0 license.

Figure S1. The extracellular-end distance differences of the inactive and active D2R structure pairs.

The heatmap shows the difference of pairwise distance among the extracellular-end between the active and inactive structures (D2R active – D2R inactive) and categorized based on various $DD_{threshold}$ (T). Red represents the active conformation have a larger distance than inactive conformation, and blue represents the inactive conformation have a larger distance than active conformation. If the category average of the difference is smaller or equal to 0.9, it is colored as white in the masked heatmaps.

bioRxiv preprint doi: https://doi.org/10.1101/2023.11.11.566699; this version posted November 14, 2023. The copyright holder for this preprint (which was not certified by peer review) is the author/funder. This article is a US Government work. It is not subject to copyright under 17 USC 105 and is also made available for use under a CC0 license.

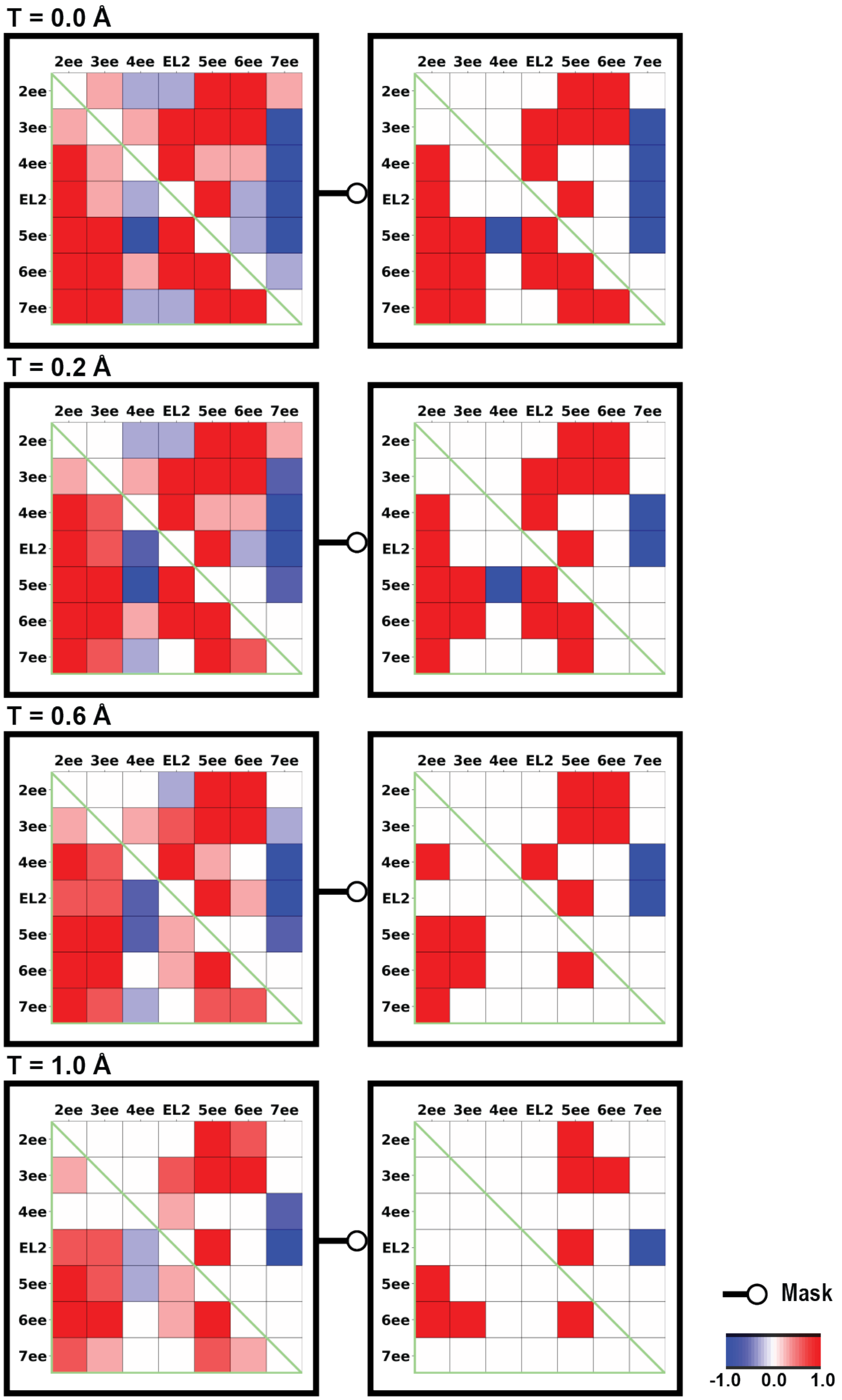

bioRxiv preprint doi: https://doi.org/10.1101/2023.11.11.566699; this version posted November 14, 2023. The copyright holder for this preprint (which was not certified by peer review) is the author/funder. This article is a US Government work. It is not subject to copyright under 17 USC 105 and is also made available for use under a CC0 license.

Figure S2. The binding site distance-difference category averages of the inactive and active D2R structure pairs.

The heatmap shows the difference of pairwise distance among the binding site residues between the active and inactive structures (D2R active – D2R inactive) and categorized based on various $DD_{threshold}$ (T). Red represents the active conformation have a larger distance than inactive conformation, and blue represents the inactive conformation have a larger distance than active conformation. If the category average of the difference is smaller or equal to 0.9, it is colored as white in the masked heatmaps.

bioRxiv preprint doi: https://doi.org/10.1101/2023.11.11.566699; this version posted November 14, 2023. The copyright holder for this preprint (which was not certified by peer review) is the author/funder. This article is a US Government work. It is not subject to copyright under 17 USC 105 and is also made available for use under a CC0 license.

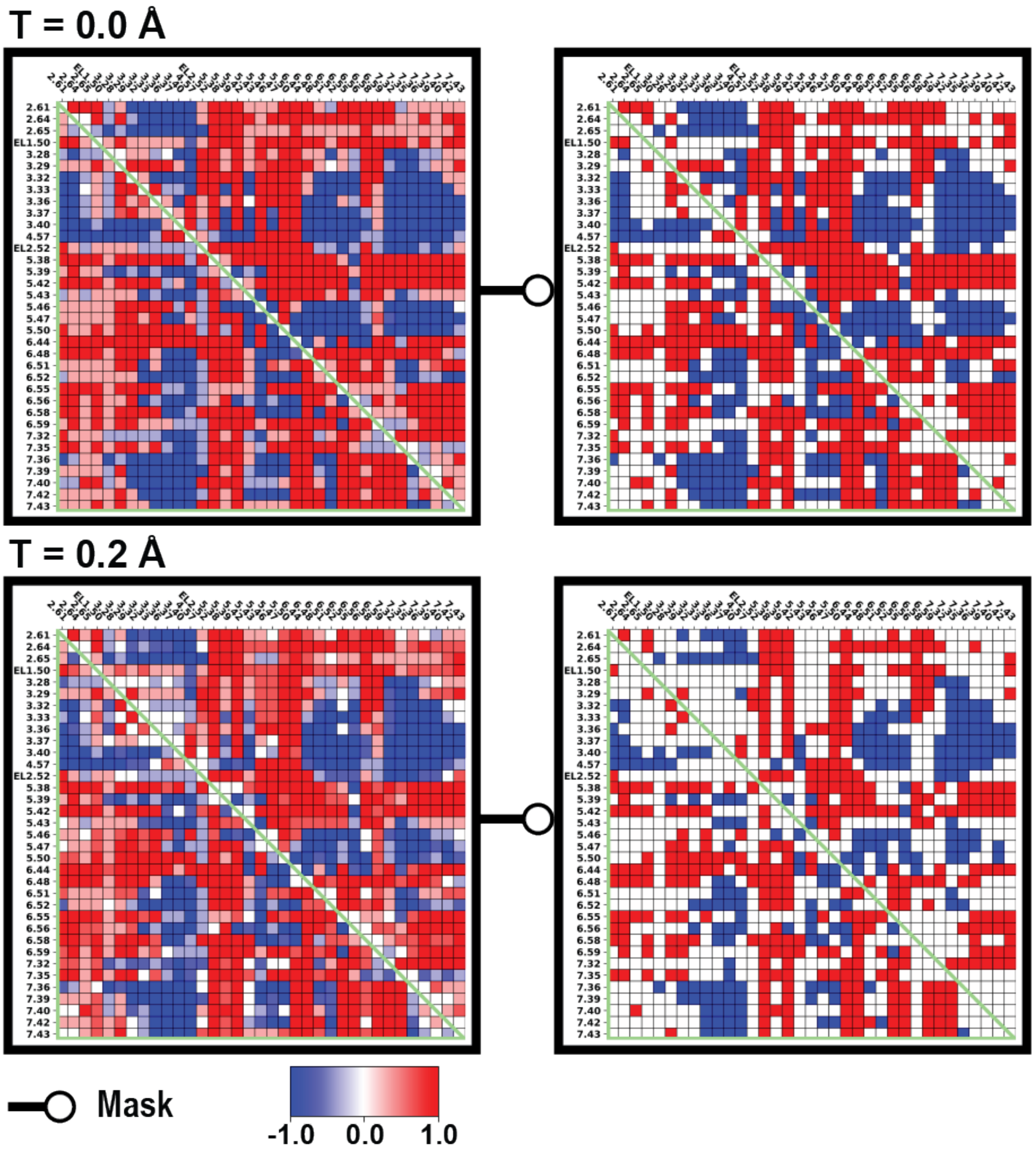

bioRxiv preprint doi: https://doi.org/10.1101/2023.11.11.566699; this version posted November 14, 2023. The copyright holder for this preprint (which was not certified by peer review) is the author/funder. This article is a US Government work. It is not subject to copyright under 17 USC 105 and is also made available for use under a CC0 license.

Figure S2. (continued)

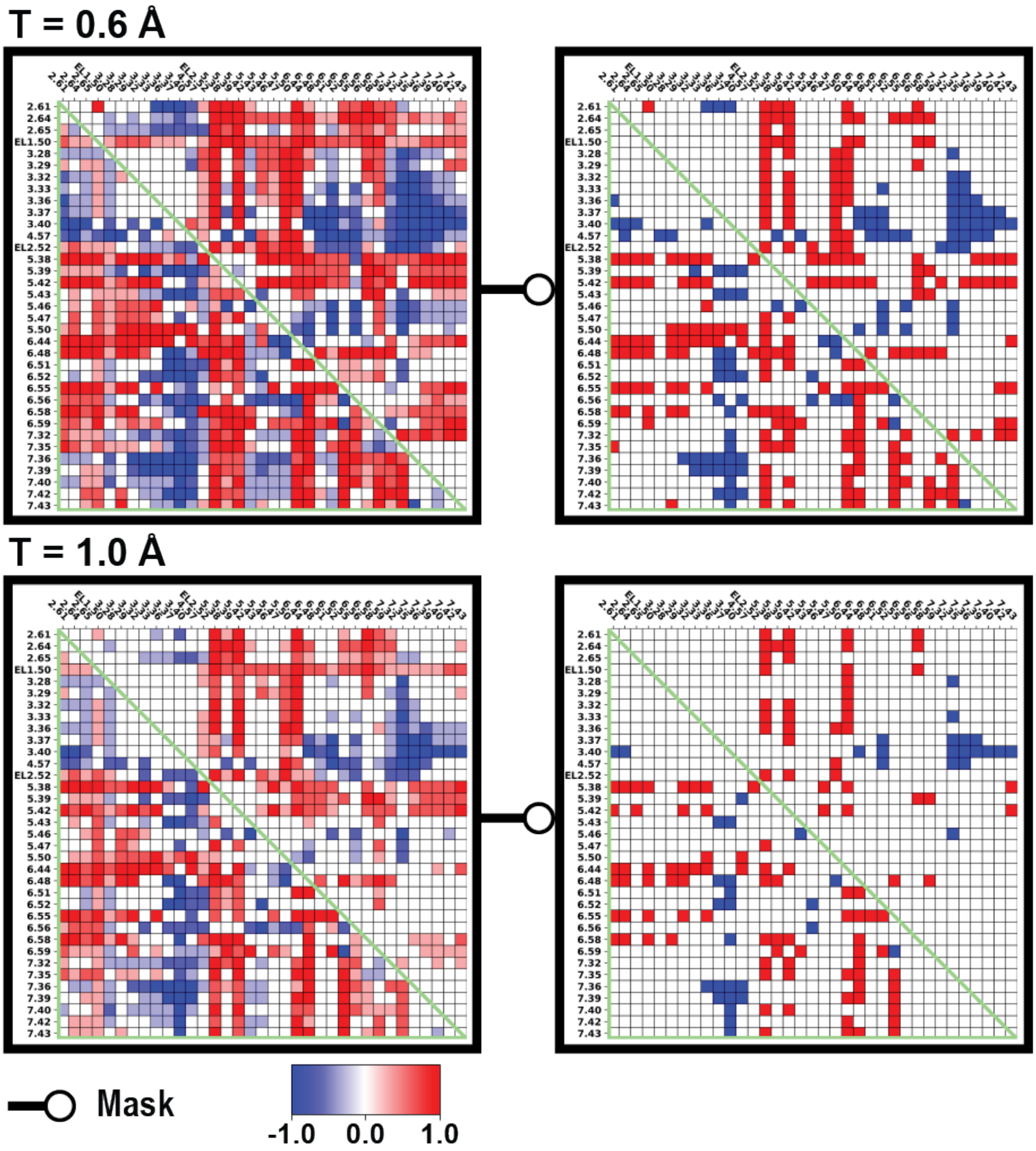

bioRxiv preprint doi: https://doi.org/10.1101/2023.11.11.566699; this version posted November 14, 2023. The copyright holder for this preprint (which was not certified by peer review) is the author/funder. This article is a US Government work. It is not subject to copyright under 17 USC 105 and is also made available for use under a CC0 license.

Figure S3. The OBS residue distance-difference category averages of the inactive and active D2R structure pairs.

The heatmap shows the difference of pairwise distance among the OBS residues between the active and inactive structures (D2R active – D2R inactive) and categorized based on various $DD_{threshold}$ (T). Red represents the active conformation have a larger distance than inactive conformation, and blue represents the inactive conformation have a larger distance than active conformation. If the category average of the difference is smaller or equal to 0.9, it is colored as white in the masked heatmaps.

bioRxiv preprint doi: https://doi.org/10.1101/2023.11.11.566699; this version posted November 14, 2023. The copyright holder for this preprint (which was not certified by peer review) is the author/funder. This article is a US Government work. It is not subject to copyright under 17 USC 105 and is also made available for use under a CC0 license.

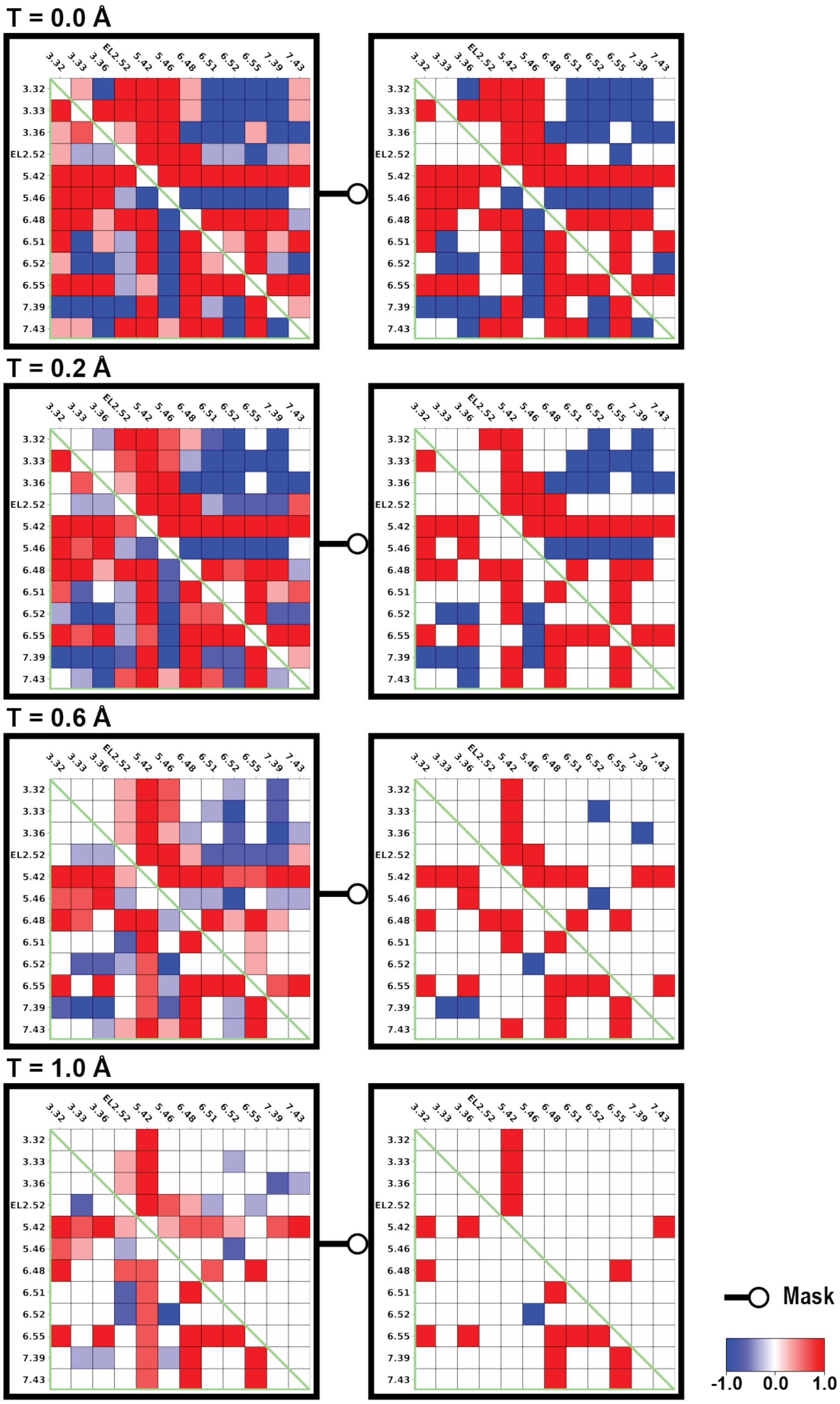

bioRxiv preprint doi: https://doi.org/10.1101/2023.11.11.566699; this version posted November 14, 2023. The copyright holder for this preprint (which was not certified by peer review) is the author/funder. This article is a US Government work. It is not subject to copyright under 17 USC 105 and is also made available for use under a CC0 license.

Figure S4. The subsegment distance-difference category averages of the inactive and active D2R structure pairs.

The heatmap shows the difference of pairwise distance among the subsegments between the active and inactive structures (D2R active – D2R inactive) and categorized based on various $DD_{threshold}$ (T). Red represents the active conformation have a larger distance than inactive conformation, and blue represents the inactive conformation have a larger distance than active conformation. If the category average of the difference is smaller or equal to 0.9, it is colored as white in the masked heatmaps.

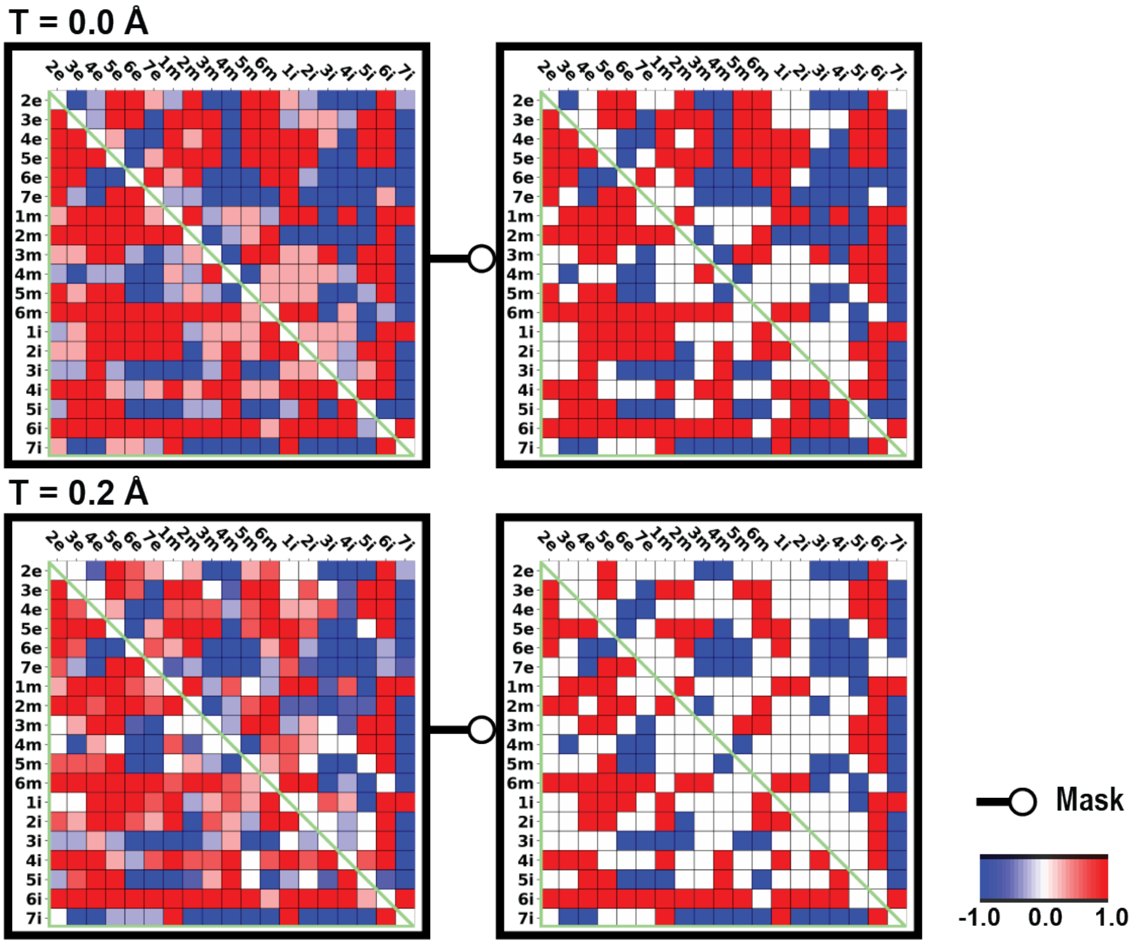

bioRxiv preprint doi: https://doi.org/10.1101/2023.11.11.566699; this version posted November 14, 2023. The copyright holder for this preprint (which was not certified by peer review) is the author/funder. This article is a US Government work. It is not subject to copyright under 17 USC 105 and is also made available for use under a CC0 license.

Figure S4 (continued)

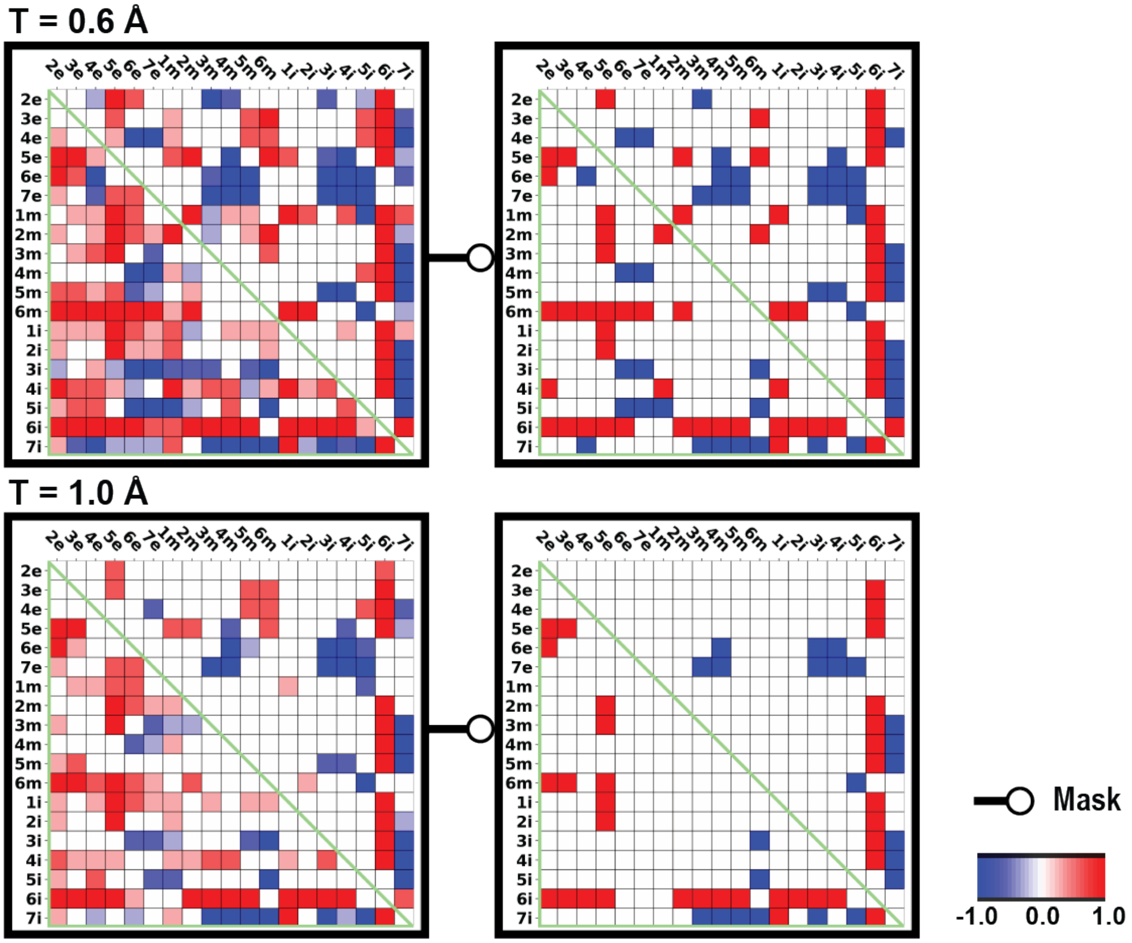

bioRxiv preprint doi: https://doi.org/10.1101/2023.11.11.566699; this version posted November 14, 2023. The copyright holder for this preprint (which was not certified by peer review) is the author/funder. This article is a US Government work. It is not subject to copyright under 17 USC 105 and is also made available for use under a CC0 license.

Figure S5. The extracellular-end distance differences of the inactive and active D3R structure pairs.

The heatmap shows the difference of pairwise distance among the extracellular-end between the active and inactive structures (D3R active – D3R inactive) and categorized based on various $DD_{threshold}$ (T). Red represents the active conformation have a larger distance than inactive conformation, and blue represents the inactive conformation have a larger distance than active conformation. If the category average of the difference is smaller or equal to 0.9, it is colored as white in the masked heatmaps.

bioRxiv preprint doi: https://doi.org/10.1101/2023.11.11.566699; this version posted November 14, 2023. The copyright holder for this preprint (which was not certified by peer review) is the author/funder. This article is a US Government work. It is not subject to copyright under 17 USC 105 and is also made available for use under a CC0 license.

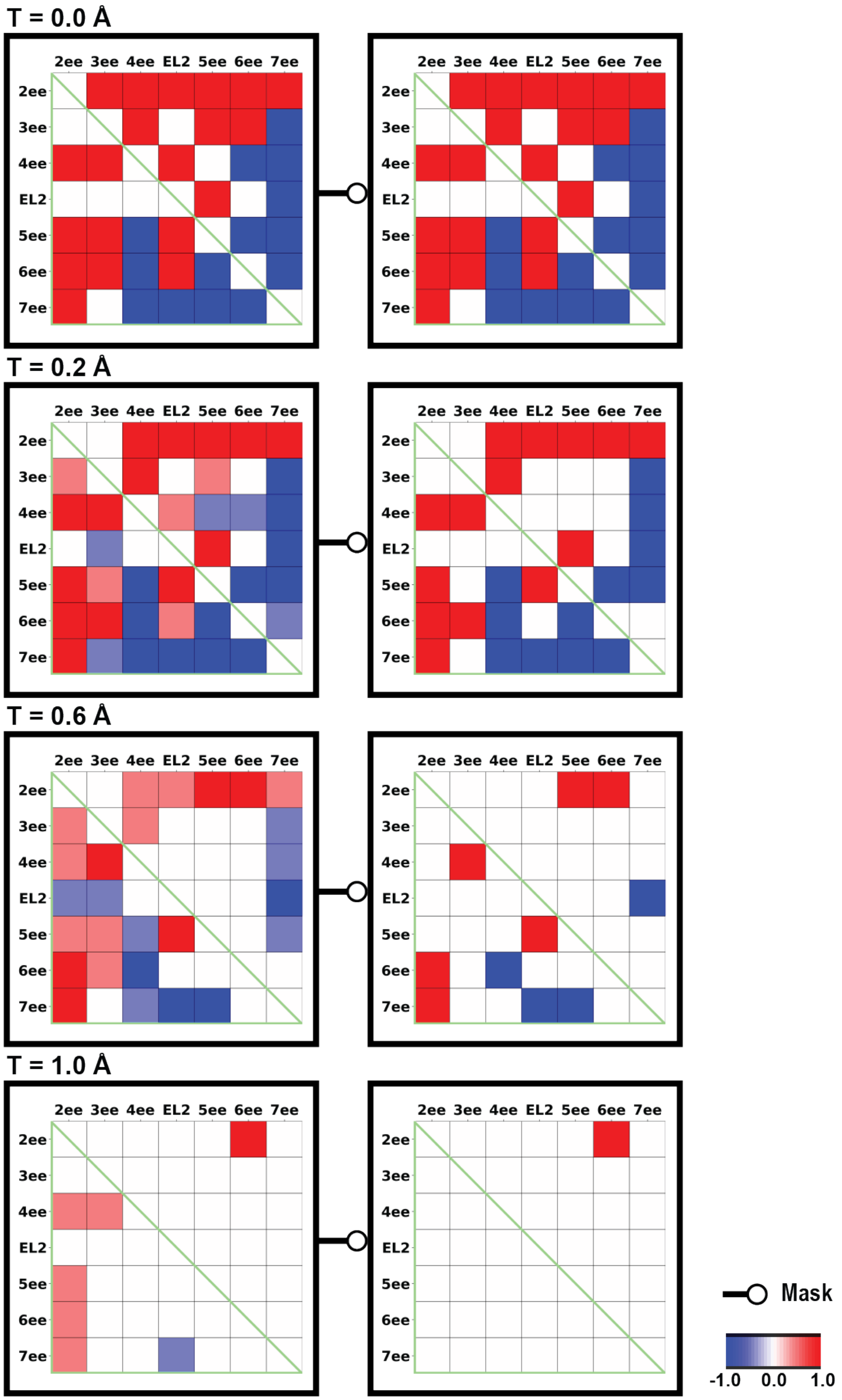

bioRxiv preprint doi: https://doi.org/10.1101/2023.11.11.566699; this version posted November 14, 2023. The copyright holder for this preprint (which was not certified by peer review) is the author/funder. This article is a US Government work. It is not subject to copyright under 17 USC 105 and is also made available for use under a CC0 license.

Figure S6. The binding site distance-difference category averages of the inactive and active D3R structure pairs.

The heatmap shows the difference of pairwise distance among the binding site residues between the active and inactive structures (D3R active – D3R inactive) and categorized based on various $DD_{threshold}$ (T). Red represents the active conformation have a larger distance than inactive conformation, and blue represents the inactive conformation have a larger distance than active conformation. If the category average of the difference is smaller or equal to 0.9, it is colored as white in the masked heatmaps.

bioRxiv preprint doi: https://doi.org/10.1101/2023.11.11.566699; this version posted November 14, 2023. The copyright holder for this preprint (which was not certified by peer review) is the author/funder. This article is a US Government work. It is not subject to copyright under 17 USC 105 and is also made available for use under a CC0 license.

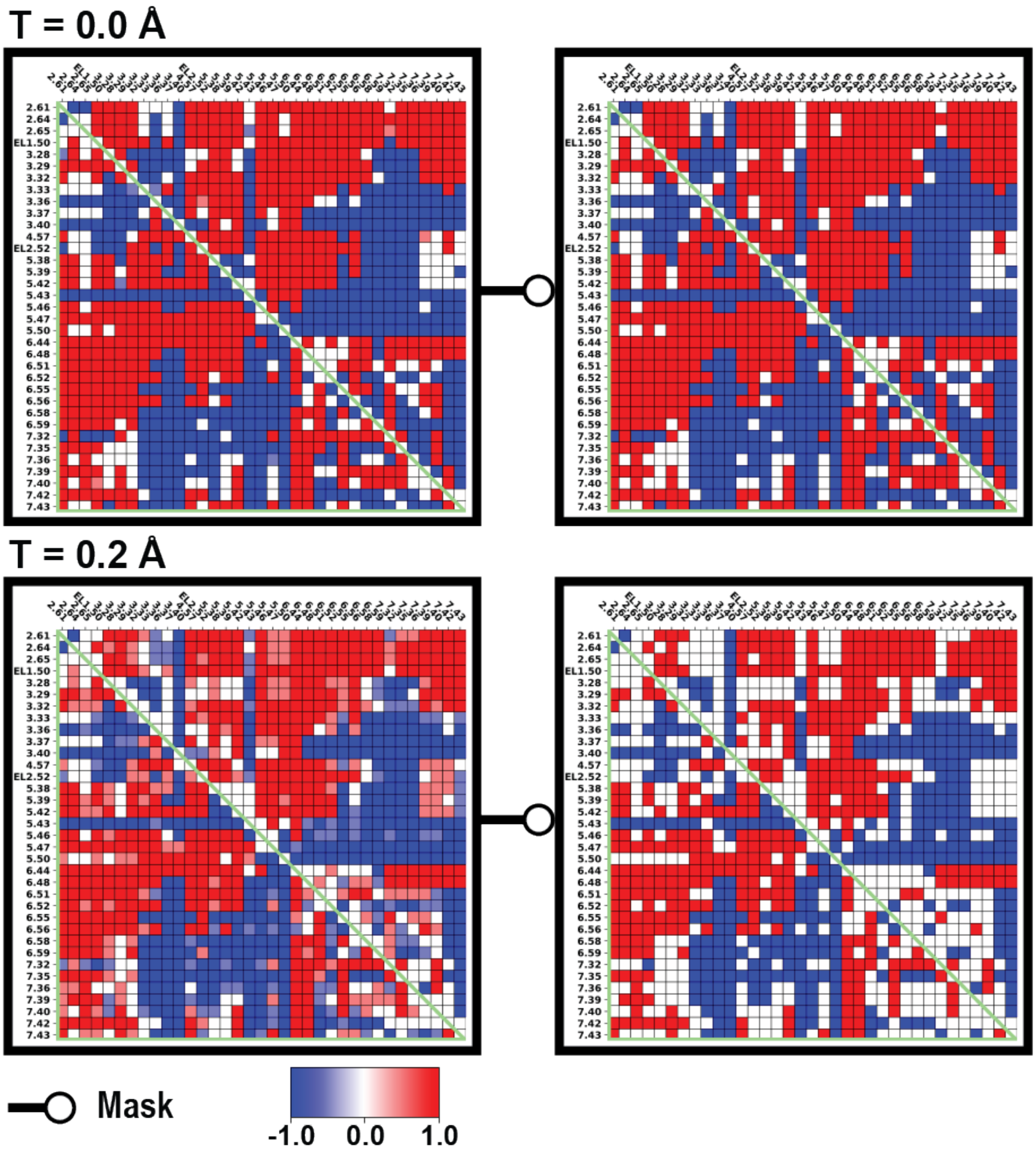

bioRxiv preprint doi: https://doi.org/10.1101/2023.11.11.566699; this version posted November 14, 2023. The copyright holder for this preprint (which was not certified by peer review) is the author/funder. This article is a US Government work. It is not subject to copyright under 17 USC 105 and is also made available for use under a CC0 license.

Figure S6. (continued)

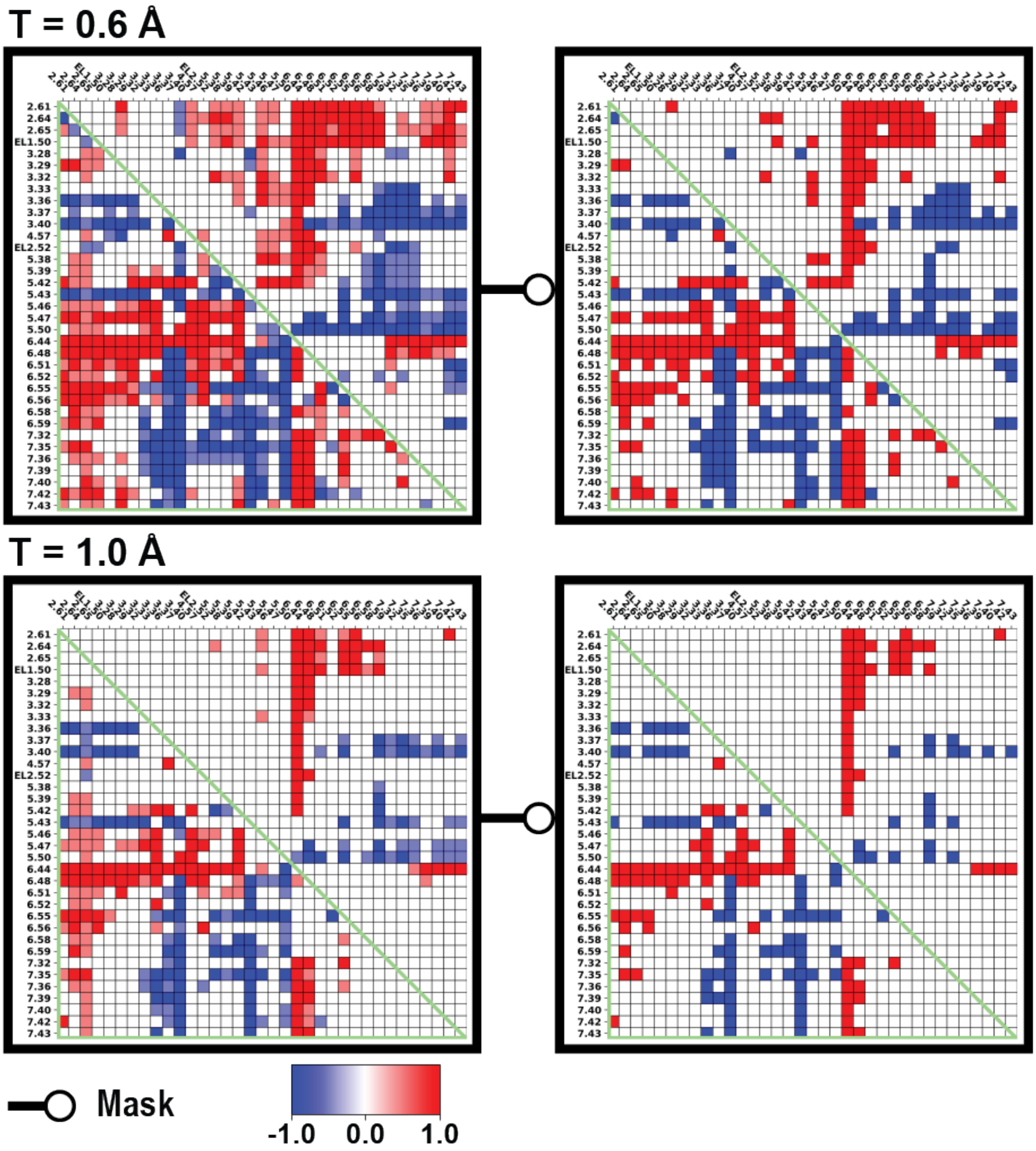

bioRxiv preprint doi: https://doi.org/10.1101/2023.11.11.566699; this version posted November 14, 2023. The copyright holder for this preprint (which was not certified by peer review) is the author/funder. This article is a US Government work. It is not subject to copyright under 17 USC 105 and is also made available for use under a CC0 license.

Figure S7. The OBS residue distance-difference category averages of the inactive and active D3R structure pairs.

The heatmap shows the difference of pairwise distance among the OBS residues between the active and inactive structures (D3R active – D3R inactive) and categorized based on various $DD_{threshold}$ (T). Red represents the active conformation have a larger distance than inactive conformation, and blue represents the inactive conformation have a larger distance than active conformation. If the category average of the difference is smaller or equal to 0.9, it is colored as white in the masked heatmaps.

bioRxiv preprint doi: https://doi.org/10.1101/2023.11.11.566699; this version posted November 14, 2023. The copyright holder for this preprint (which was not certified by peer review) is the author/funder. This article is a US Government work. It is not subject to copyright under 17 USC 105 and is also made available for use under a CC0 license.

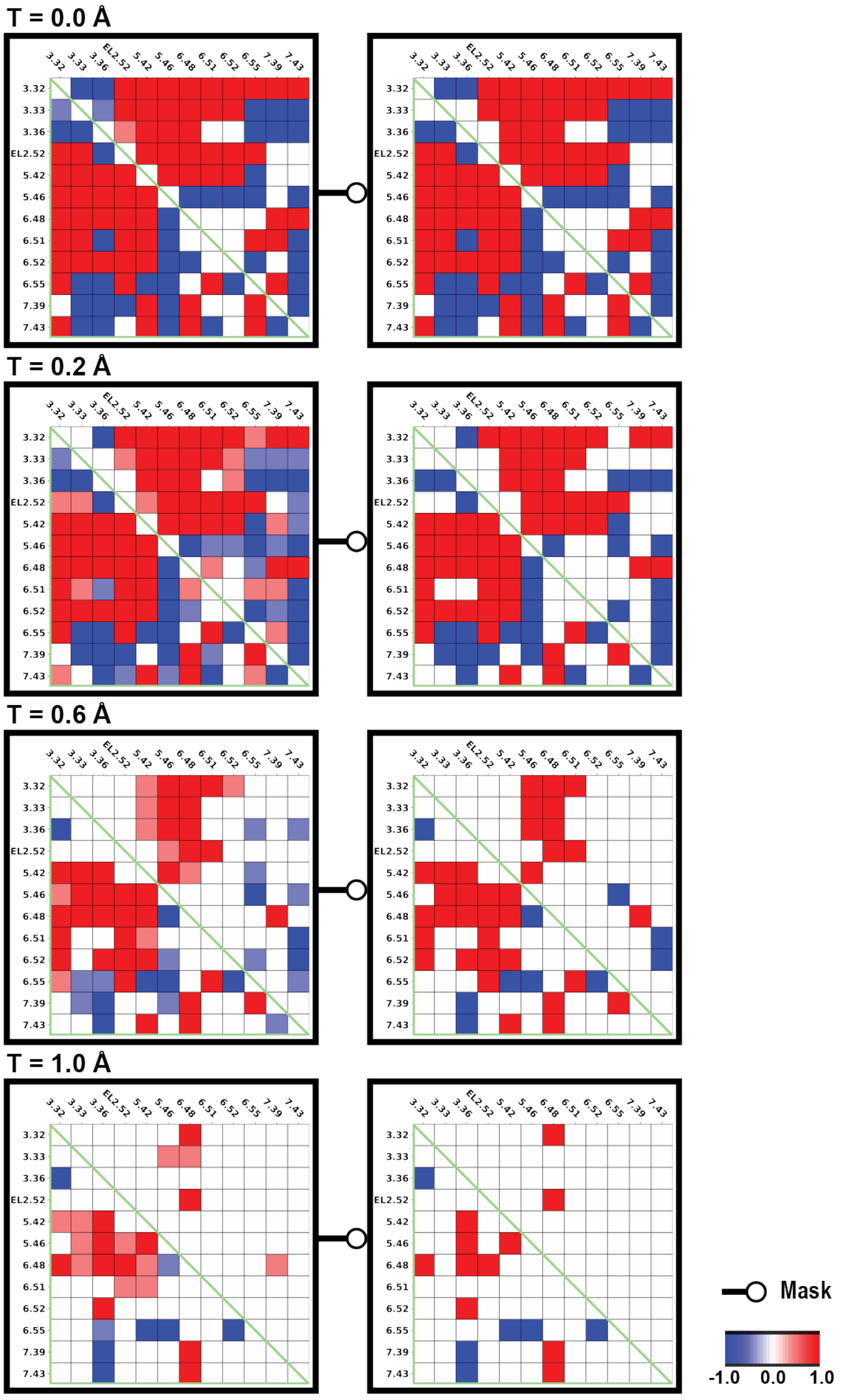

bioRxiv preprint doi: https://doi.org/10.1101/2023.11.11.566699; this version posted November 14, 2023. The copyright holder for this preprint (which was not certified by peer review) is the author/funder. This article is a US Government work. It is not subject to copyright under 17 USC 105 and is also made available for use under a CC0 license.

Figure S8. The subsegment distance-difference category averages of the inactive and active D3R structure pairs.

The heatmap shows the difference of pairwise distance among the subsegments between the active and inactive structures (D3R active – D3R inactive) and categorized based on various $DD_{threshold}$ (T). Red represents the active conformation have a larger distance than inactive conformation, and blue represents the inactive conformation have a larger distance than active conformation. If the category average of the difference is smaller or equal to 0.9, it is colored as white in the masked heatmaps.

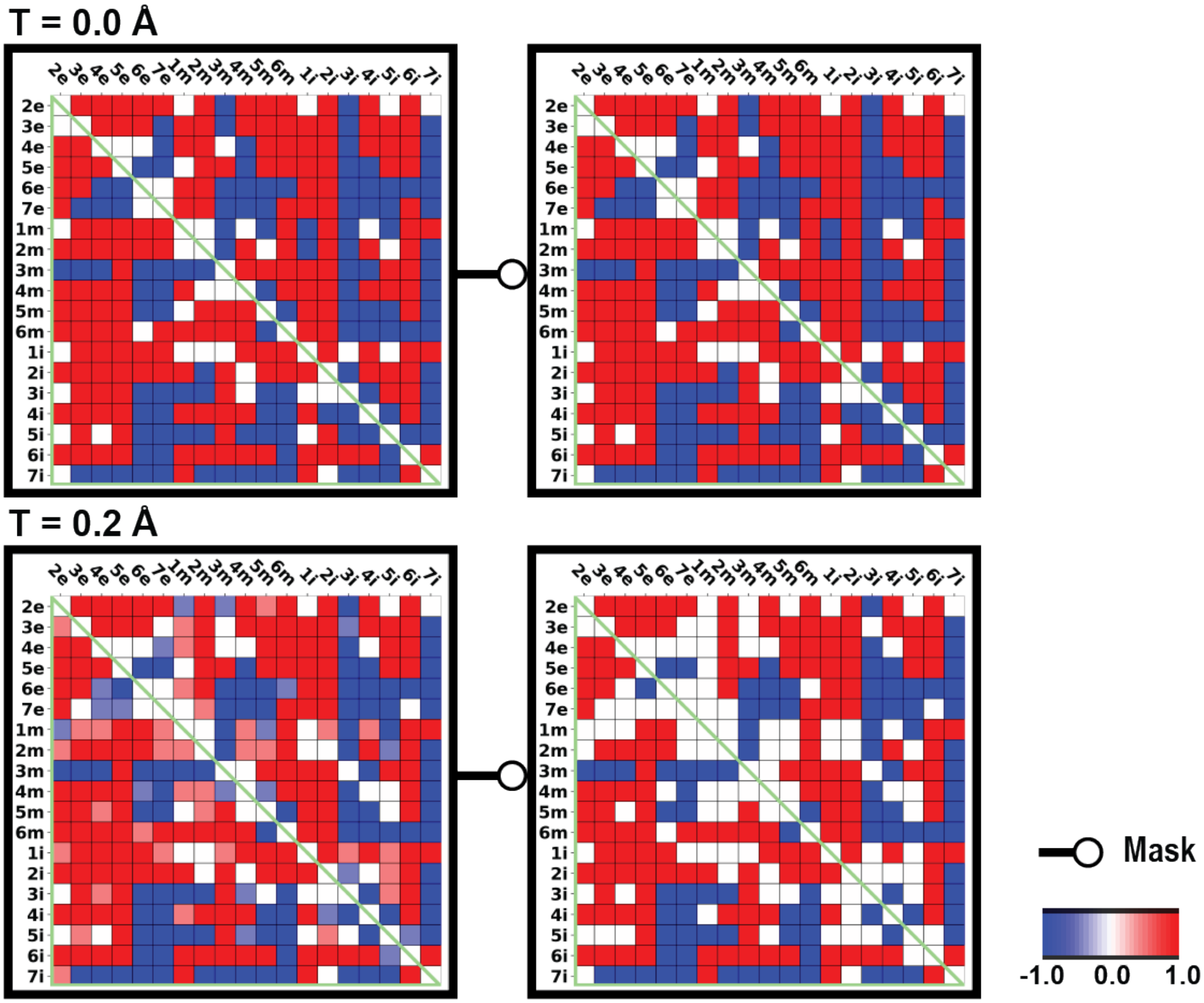

bioRxiv preprint doi: https://doi.org/10.1101/2023.11.11.566699; this version posted November 14, 2023. The copyright holder for this preprint (which was not certified by peer review) is the author/funder. This article is a US Government work. It is not subject to copyright under 17 USC 105 and is also made available for use under a CC0 license.

Figure S8 (continued)

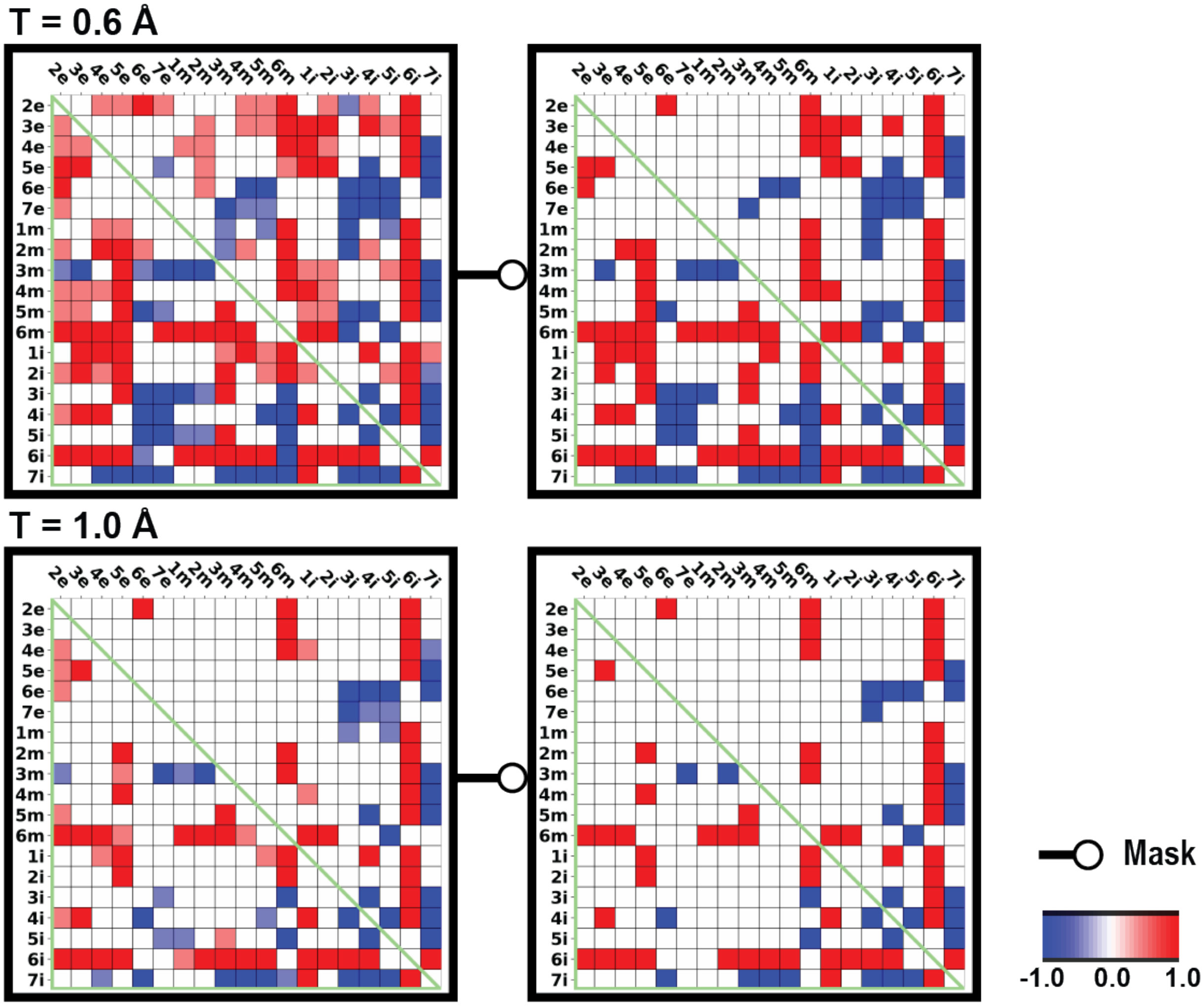

bioRxiv preprint doi: https://doi.org/10.1101/2023.11.11.566699; this version posted November 14, 2023. The copyright holder for this preprint (which was not certified by peer review) is the author/funder. This article is a US Government work. It is not subject to copyright under 17 USC 105 and is also made available for use under a CC0 license.

Figure S9. The extracellular-end distance differences of the inactive and active D2R and D3R structure pairs.

The heatmap shows the difference of pairwise distance among the extracellular-end between the active and inactive structures (active – inactive) and categorized based on various $DD_{threshold}$ (T). Red represents the active conformation have a larger distance than inactive conformation, and blue represents the inactive conformation have a larger distance than active conformation. If the category average of the difference is smaller or equal to 0.9, it is colored as white in the masked heatmaps.

bioRxiv preprint doi: https://doi.org/10.1101/2023.11.11.566699; this version posted November 14, 2023. The copyright holder for this preprint (which was not certified by peer review) is the author/funder. This article is a US Government work. It is not subject to copyright under 17 USC 105 and is also made available for use under a CC0 license.

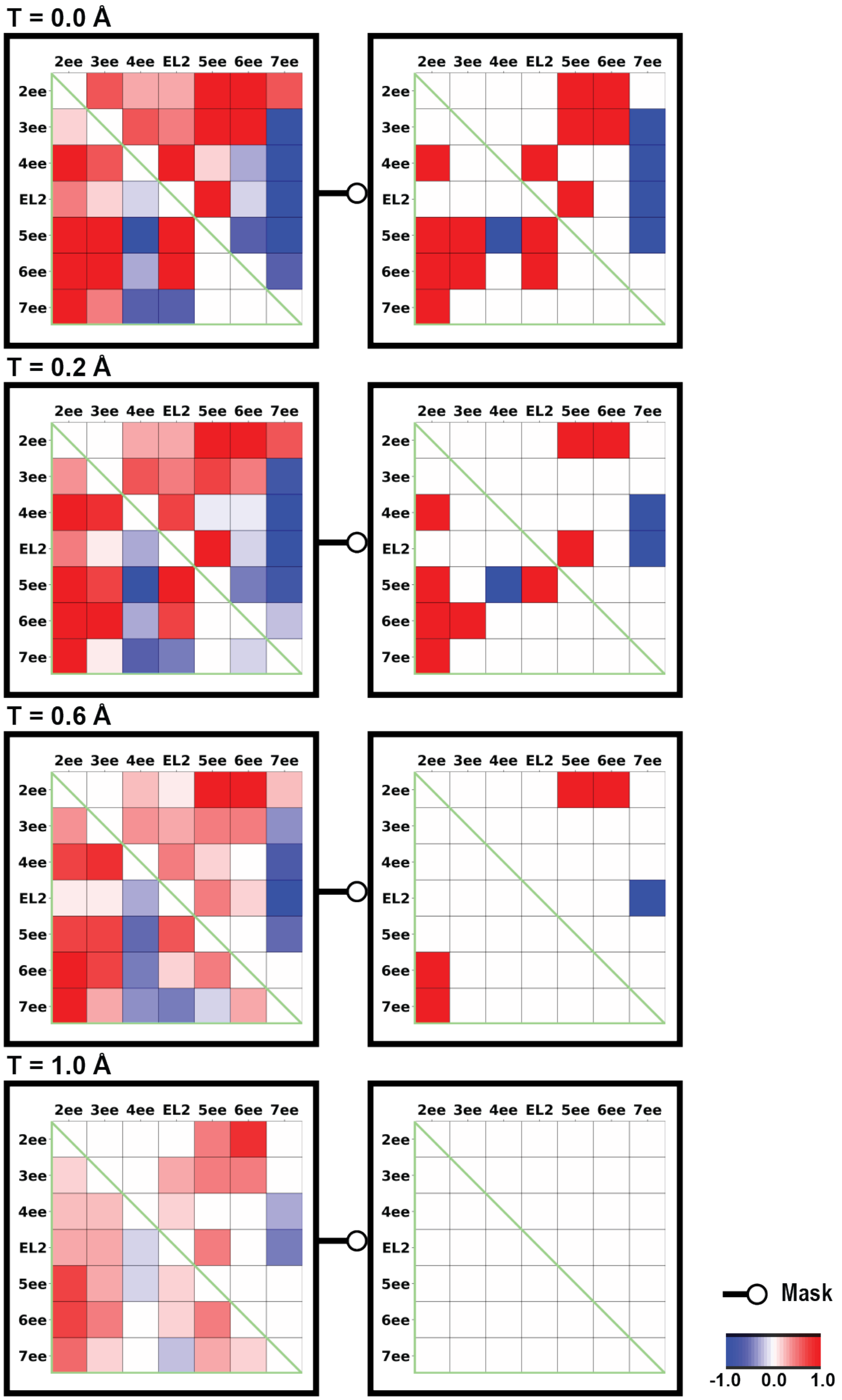

bioRxiv preprint doi: https://doi.org/10.1101/2023.11.11.566699; this version posted November 14, 2023. The copyright holder for this preprint (which was not certified by peer review) is the author/funder. This article is a US Government work. It is not subject to copyright under 17 USC 105 and is also made available for use under a CC0 license.

Figure S10. The binding site distance-difference category averages of the inactive and active D2R and D3R structure pairs.

The heatmap shows the difference of pairwise distance among the binding site residues between the active and inactive structures (active –inactive) and categorized based on various $DD_{threshold}$ (T). Red represents the active conformation have a larger distance than inactive conformation, and blue represents the inactive conformation have a larger distance than active conformation. If the category average of the difference is smaller or equal to 0.9, it is colored as white in the masked heatmaps.

bioRxiv preprint doi: https://doi.org/10.1101/2023.11.11.566699; this version posted November 14, 2023. The copyright holder for this preprint (which was not certified by peer review) is the author/funder. This article is a US Government work. It is not subject to copyright under 17 USC 105 and is also made available for use under a CC0 license.

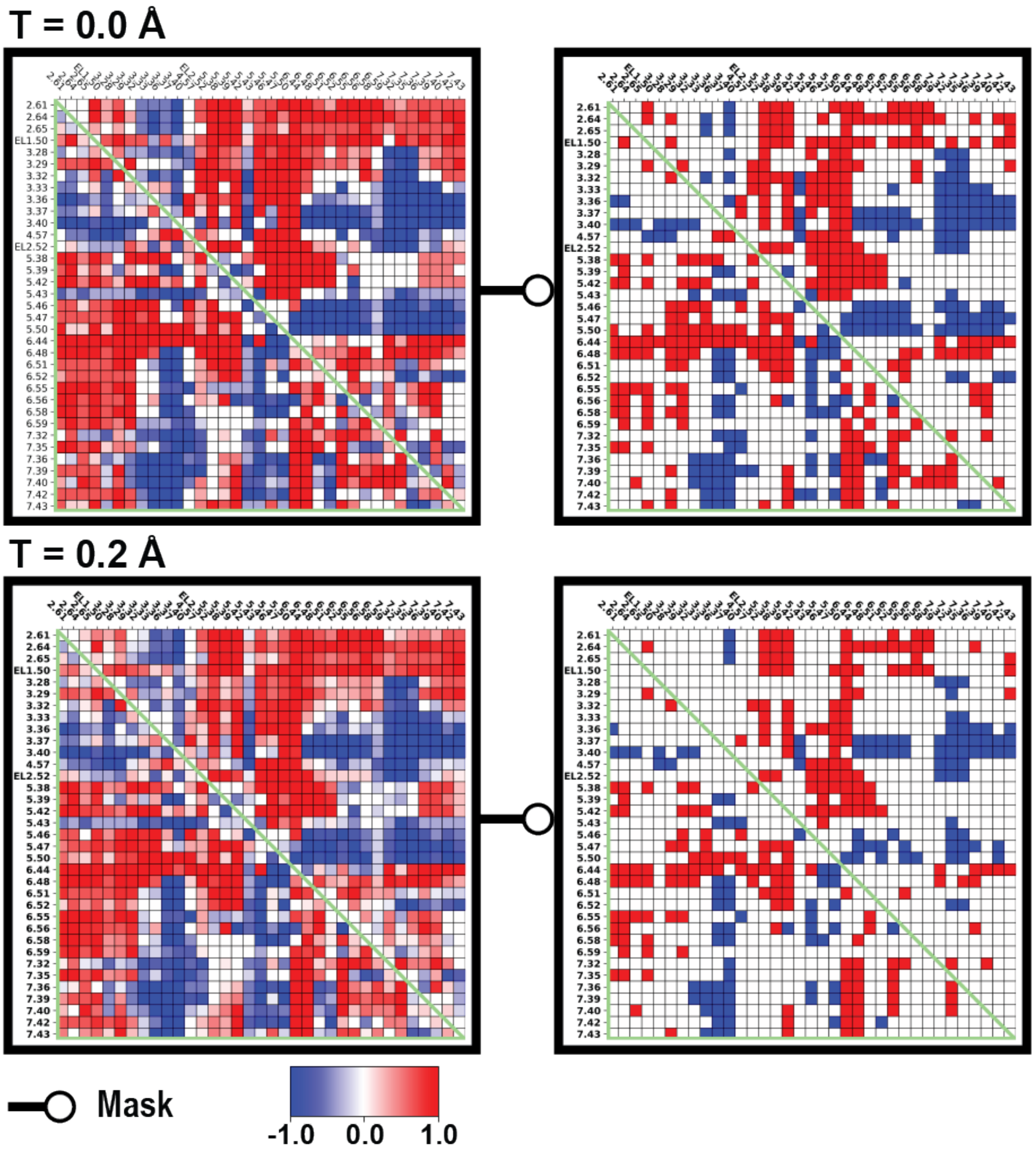

bioRxiv preprint doi: https://doi.org/10.1101/2023.11.11.566699; this version posted November 14, 2023. The copyright holder for this preprint (which was not certified by peer review) is the author/funder. This article is a US Government work. It is not subject to copyright under 17 USC 105 and is also made available for use under a CC0 license.

Figure S10. (continued)

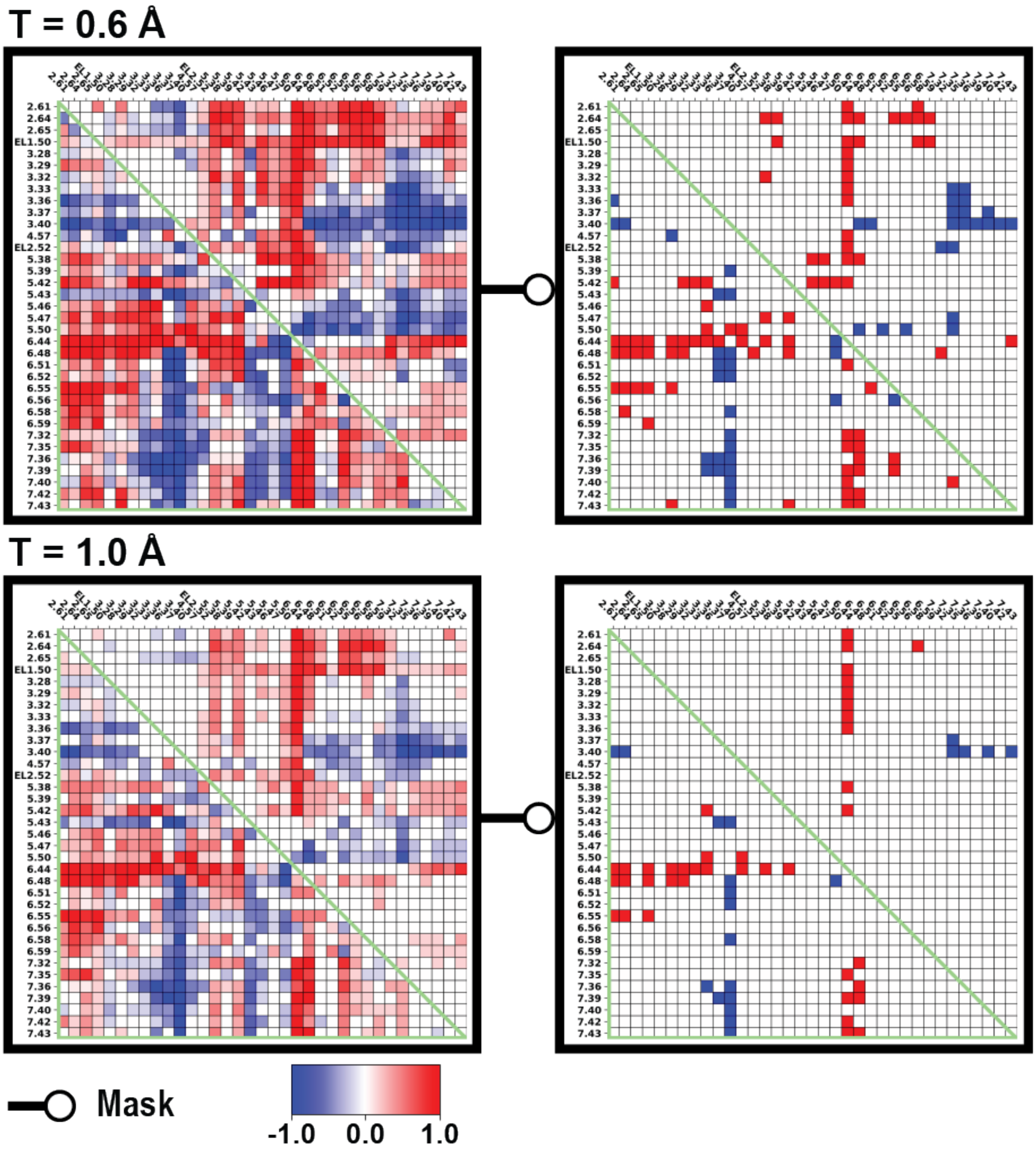

bioRxiv preprint doi: https://doi.org/10.1101/2023.11.11.566699; this version posted November 14, 2023. The copyright holder for this preprint (which was not certified by peer review) is the author/funder. This article is a US Government work. It is not subject to copyright under 17 USC 105 and is also made available for use under a CC0 license.

Figure S11. The OBS residue distance-difference category averages of the inactive and active D2R and D3R structure pairs.

The heatmap shows the difference of pairwise distance among the OBS residues between the active and inactive structures (active – inactive) and categorized based on various $DD_{threshold}$ (T). Red represents the active conformation have a larger distance than inactive conformation, and blue represents the inactive conformation have a larger distance than active conformation. If the category average of the difference is smaller or equal to 0.9, it is colored as white in the masked heatmaps.

bioRxiv preprint doi: https://doi.org/10.1101/2023.11.11.566699; this version posted November 14, 2023. The copyright holder for this preprint (which was not certified by peer review) is the author/funder. This article is a US Government work. It is not subject to copyright under 17 USC 105 and is also made available for use under a CC0 license.

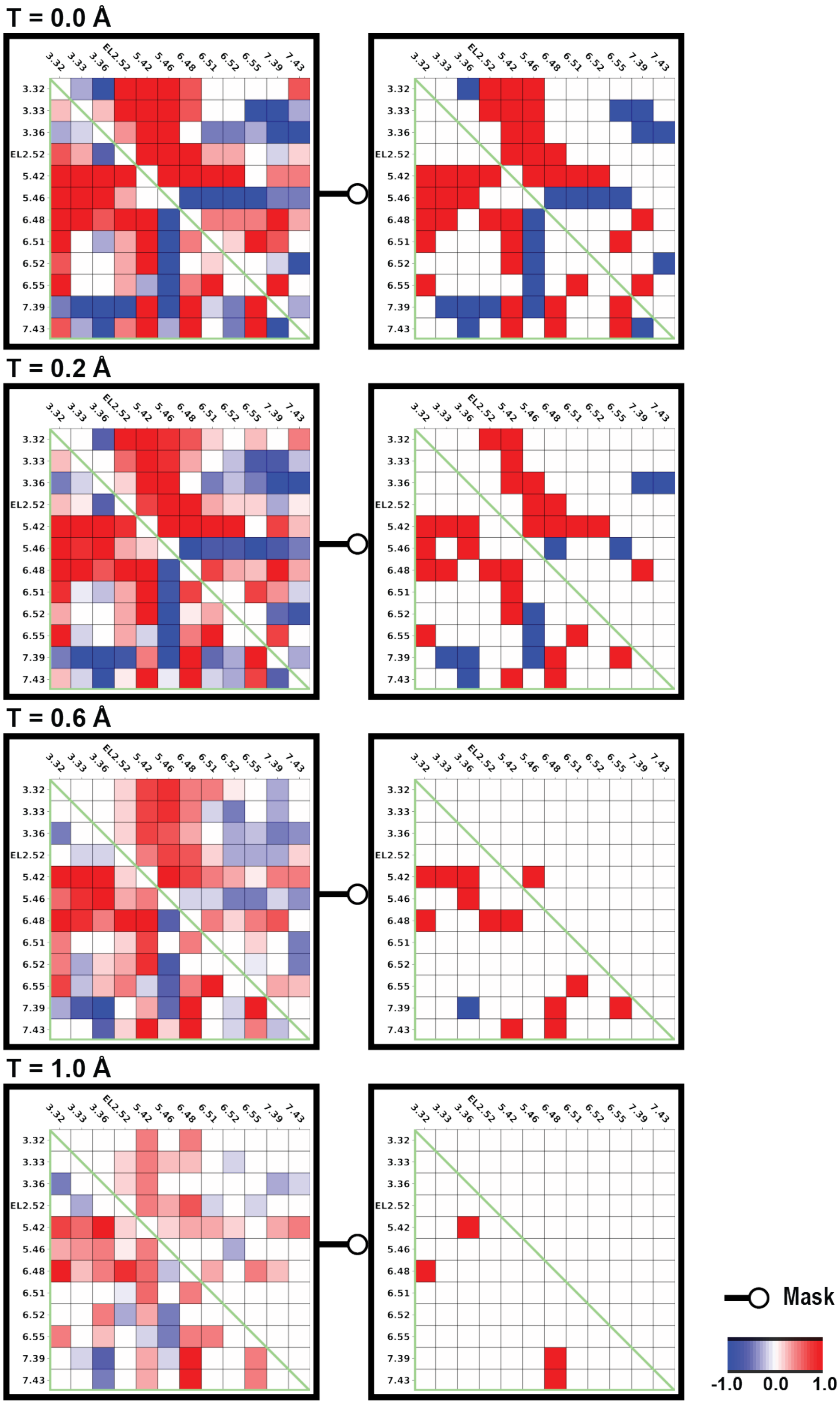

bioRxiv preprint doi: https://doi.org/10.1101/2023.11.11.566699; this version posted November 14, 2023. The copyright holder for this preprint (which was not certified by peer review) is the author/funder. This article is a US Government work. It is not subject to copyright under 17 USC 105 and is also made available for use under a CC0 license.

Figure S12. The subsegment distance-difference category averages of the inactive and active D2R and D3R structure pairs.

The heatmap shows the difference of pairwise distance among the subsegments between the active and inactive structures (active – inactive) and categorized based on various $DD_{threshold}$ (T). Red represents the active conformation have a larger distance than inactive conformation, and blue represents the inactive conformation have a larger distance than active conformation. If the category average of the difference is smaller or equal to 0.9, it is colored as white in the masked heatmaps.

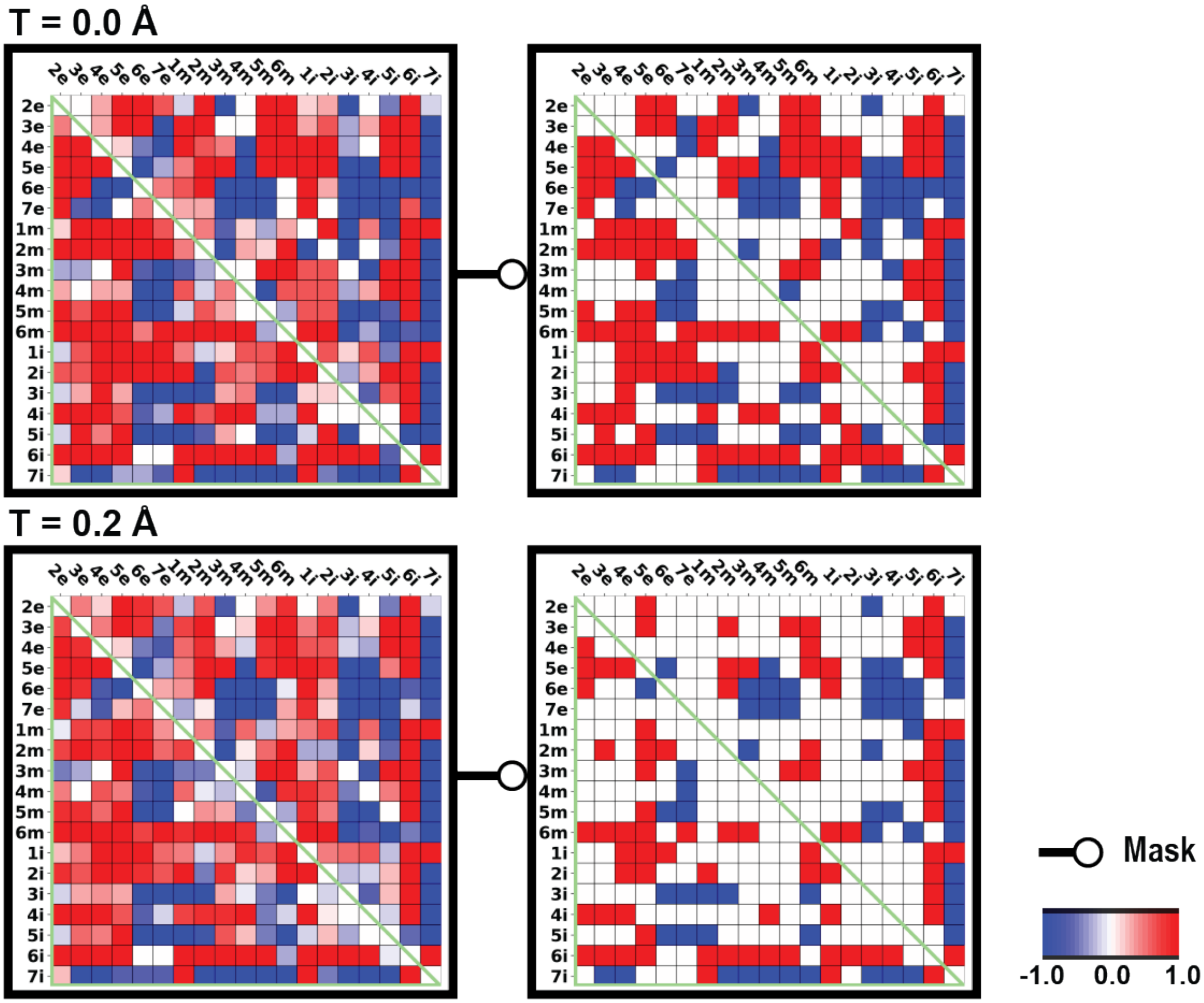

bioRxiv preprint doi: https://doi.org/10.1101/2023.11.11.566699; this version posted November 14, 2023. The copyright holder for this preprint (which was not certified by peer review) is the author/funder. This article is a US Government work. It is not subject to copyright under 17 USC 105 and is also made available for use under a CC0 license.

Figure S12 (continued)

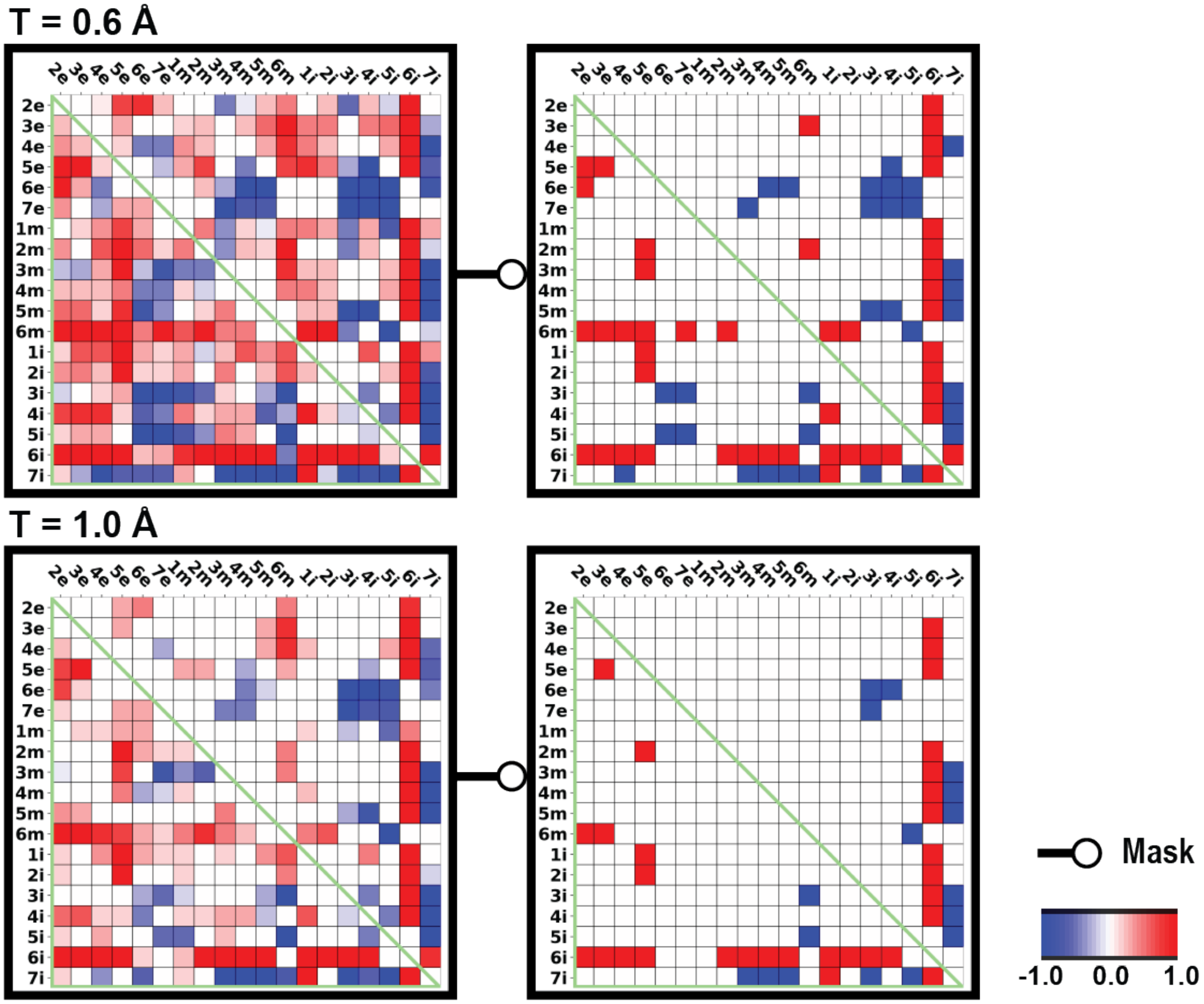

bioRxiv preprint doi: https://doi.org/10.1101/2023.11.11.566699; this version posted November 14, 2023. The copyright holder for this preprint (which was not certified by peer review) is the author/funder. This article is a US Government work. It is not subject to copyright under 17 USC 105 and is also made available for use under a CC0 license.

Figure S13. The binding site distance-difference category averages of the comparison between the inactive D3R and D2R structures.

The heatmap shows the difference of pairwise distance among the binding site residues between the D3R and D2R using their inactive structures (D3R – D2R) and categorized based on various $DD_{threshold}$ (T). Green represents the D3R conformation have a larger distance than D2R conformation, and brown represents the D2R conformation have a larger distance than D3R conformation. If the category average of the difference is smaller or equal to 0.9, it is colored as white in the masked heatmaps.

bioRxiv preprint doi: https://doi.org/10.1101/2023.11.11.566699; this version posted November 14, 2023. The copyright holder for this preprint (which was not certified by peer review) is the author/funder. This article is a US Government work. It is not subject to copyright under 17 USC 105 and is also made available for use under a CC0 license.

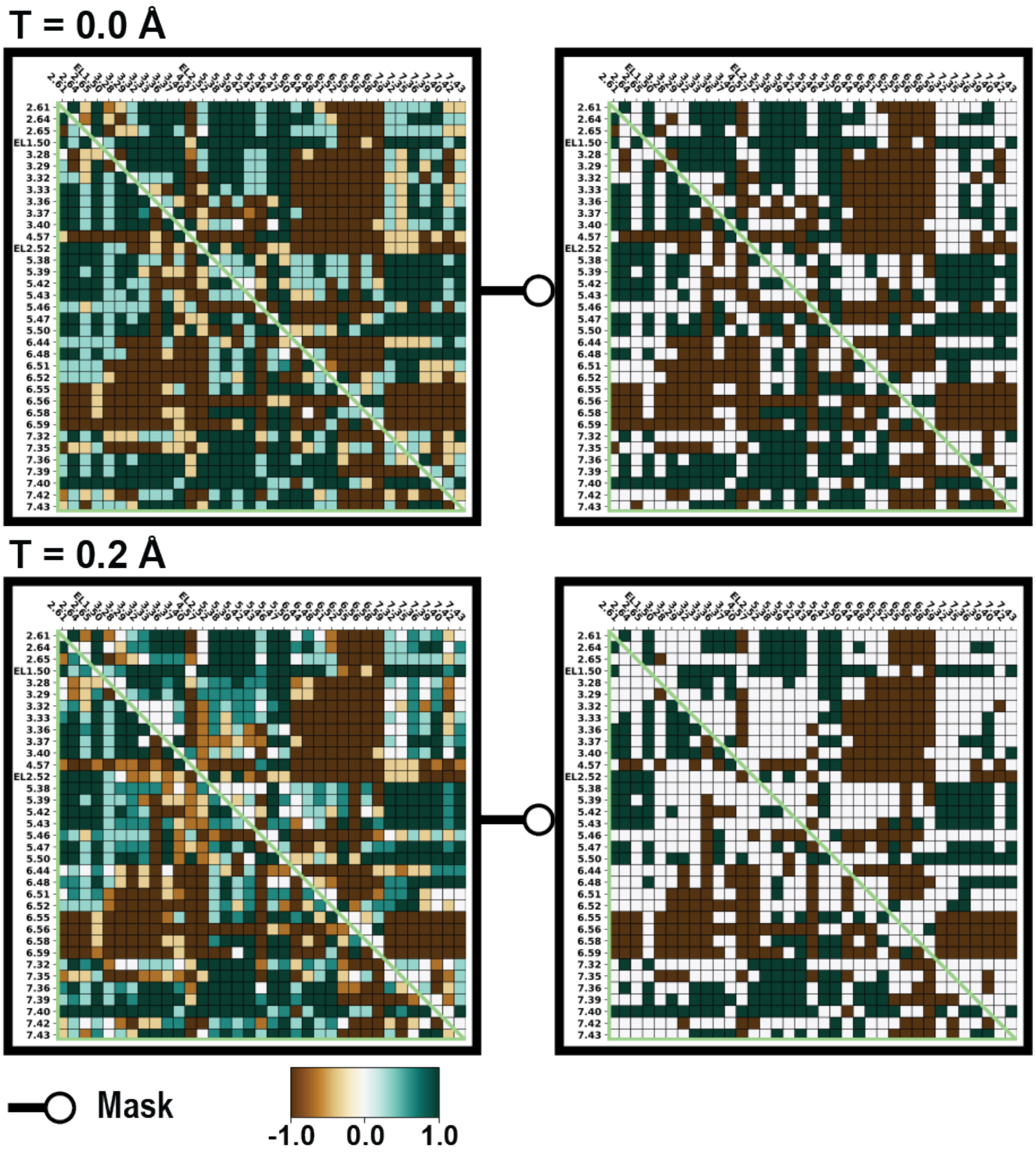

bioRxiv preprint doi: https://doi.org/10.1101/2023.11.11.566699; this version posted November 14, 2023. The copyright holder for this preprint (which was not certified by peer review) is the author/funder. This article is a US Government work. It is not subject to copyright under 17 USC 105 and is also made available for use under a CC0 license.

Figure S13. (continued)

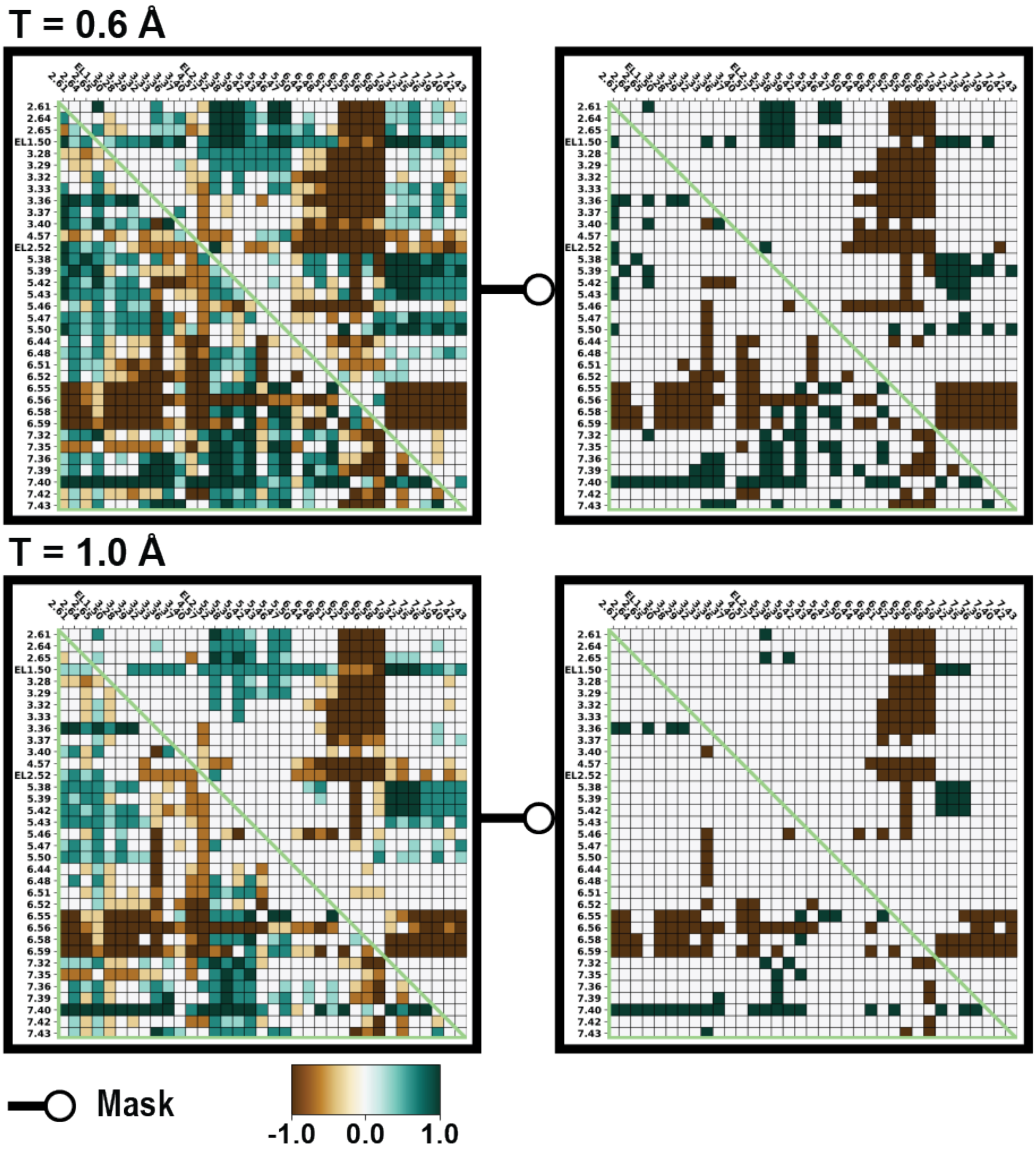

bioRxiv preprint doi: https://doi.org/10.1101/2023.11.11.566699; this version posted November 14, 2023. The copyright holder for this preprint (which was not certified by peer review) is the author/funder. This article is a US Government work. It is not subject to copyright under 17 USC 105 and is also made available for use under a CC0 license.

Figure S14. The OBS residue distance-difference category averages of the comparison between the inactive D3R and D2R structures.

The heatmap shows the difference of pairwise distance among the OBS residues between the D3R and D2R using their inactive structures (D3R – D2R) and categorized based on various $DD_{threshold}$ (T). Green represents the D3R conformation have a larger distance than D2R conformation, and brown represents the D2R conformation have a larger distance than D3R conformation. If the category average of the difference is smaller or equal to 0.9, it is colored as white in the masked heatmaps.

bioRxiv preprint doi: https://doi.org/10.1101/2023.11.11.566699; this version posted November 14, 2023. The copyright holder for this preprint (which was not certified by peer review) is the author/funder. This article is a US Government work. It is not subject to copyright under 17 USC 105 and is also made available for use under a CC0 license.

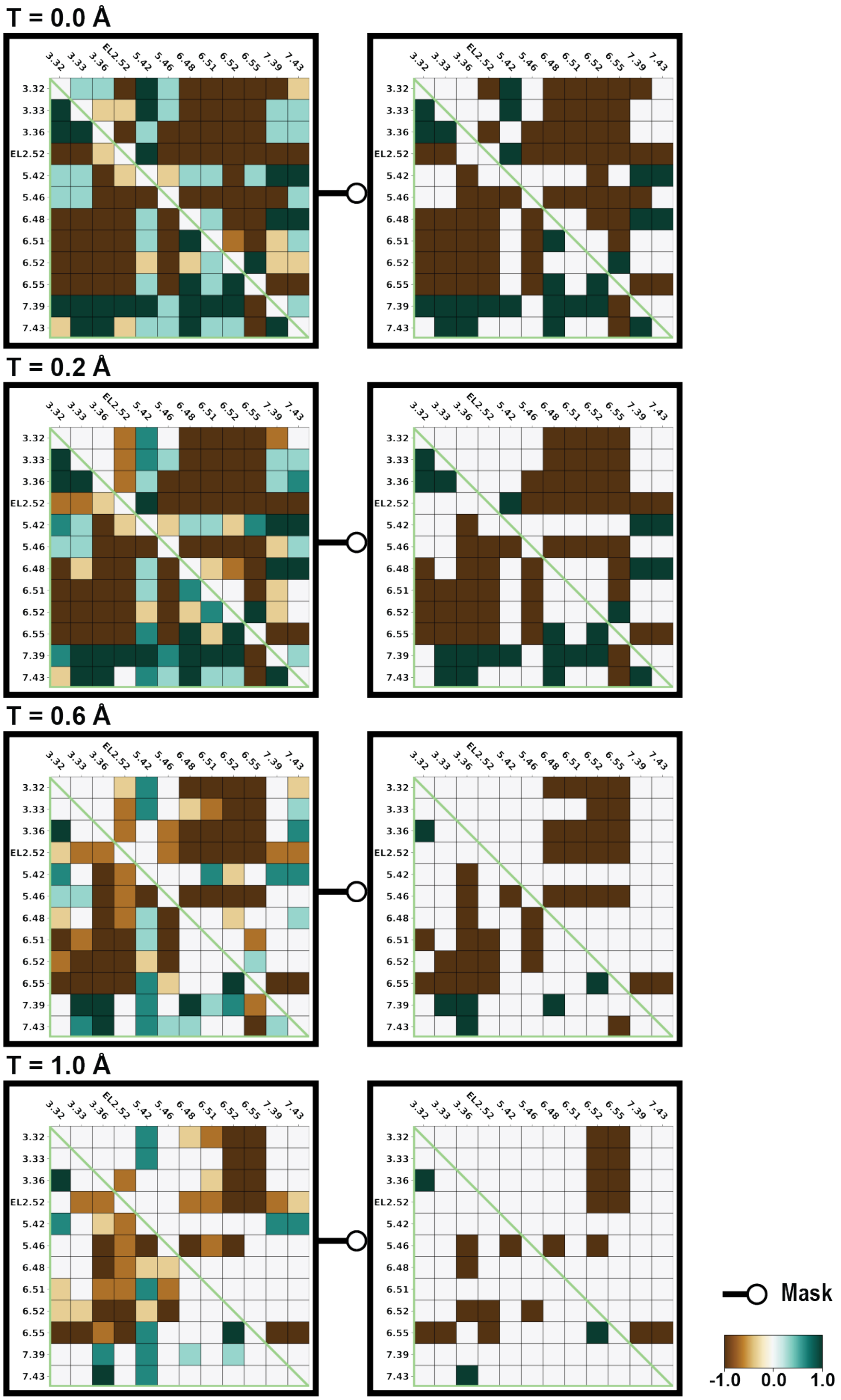

bioRxiv preprint doi: https://doi.org/10.1101/2023.11.11.566699; this version posted November 14, 2023. The copyright holder for this preprint (which was not certified by peer review) is the author/funder. This article is a US Government work. It is not subject to copyright under 17 USC 105 and is also made available for use under a CC0 license.

Figure S15. The subsegment distance-difference category averages of the comparison between the inactive D3R and D2R structures.

The heatmap shows the difference of pairwise distance among the subsegments between the D3R and D2R using their inactive structures (D3R – D2R) and categorized based on various $DD_{threshold}$ (T). Green represents the D3R conformation have a larger distance than D2R conformation, and brown represents the D2R conformation have a larger distance than D3R conformation. If the category average of the difference is smaller or equal to 0.9, it is colored as white in the masked heatmaps.

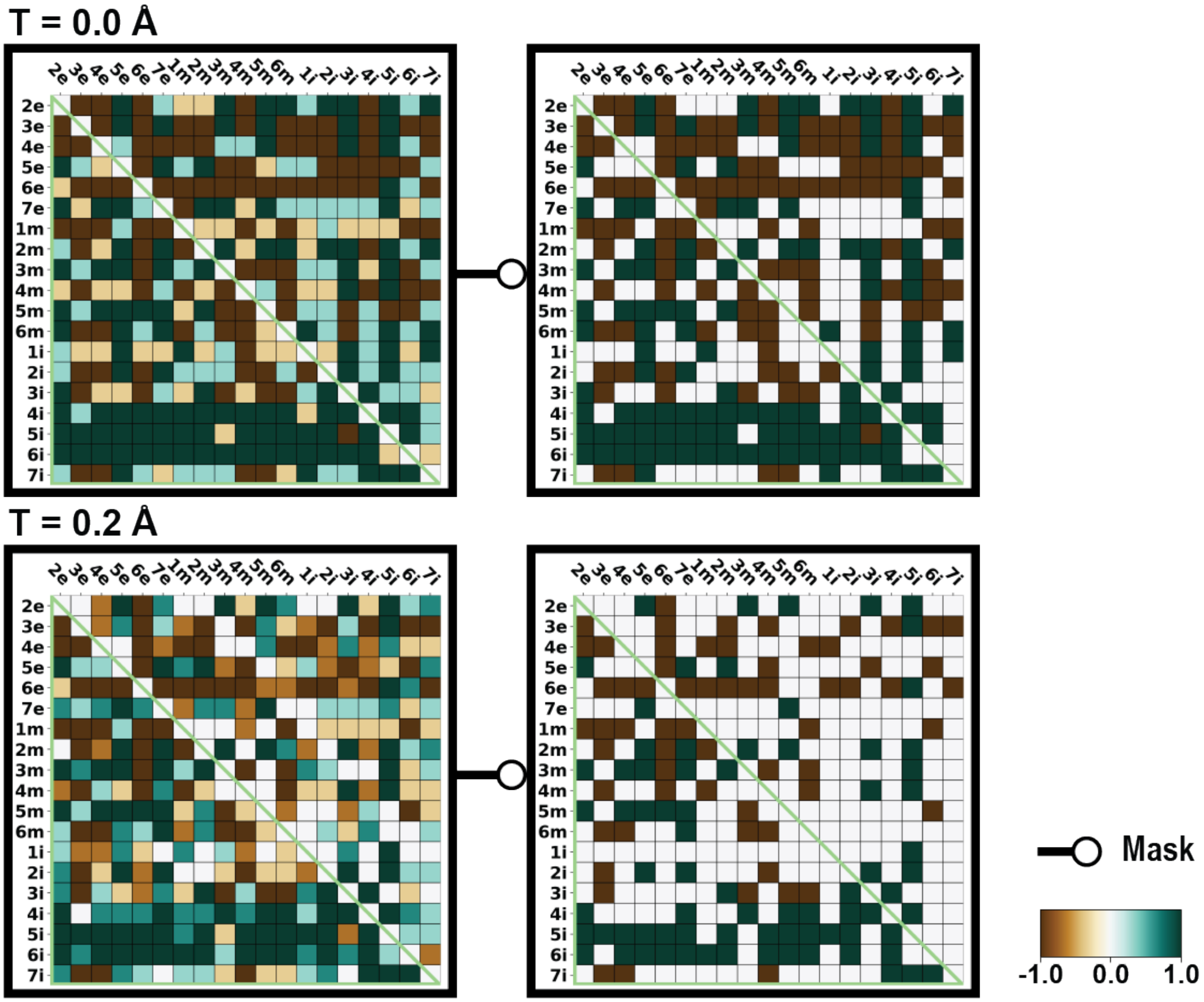

bioRxiv preprint doi: https://doi.org/10.1101/2023.11.11.566699; this version posted November 14, 2023. The copyright holder for this preprint (which was not certified by peer review) is the author/funder. This article is a US Government work. It is not subject to copyright under 17 USC 105 and is also made available for use under a CC0 license.

Figure S15. (continued)

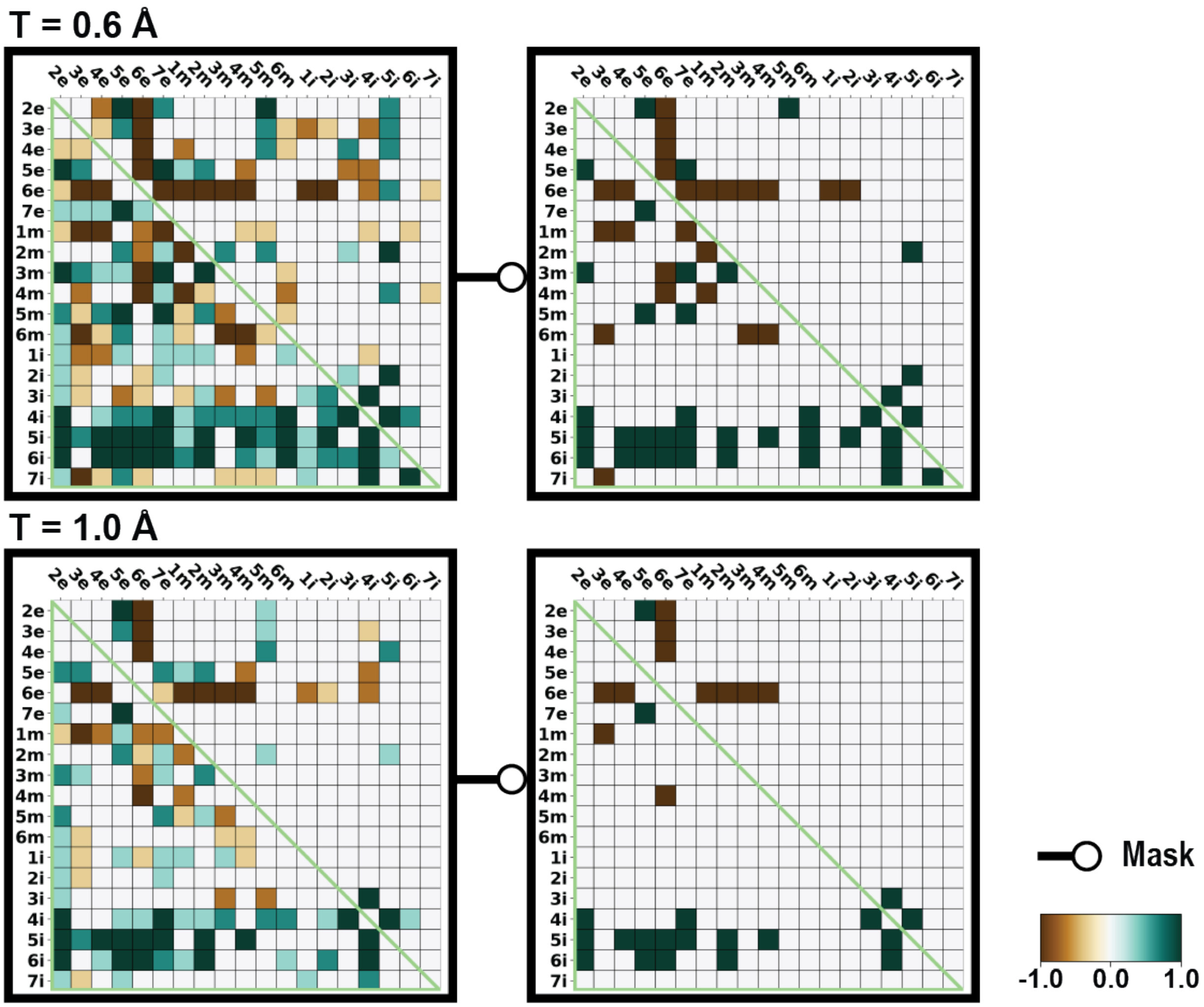

bioRxiv preprint doi: https://doi.org/10.1101/2023.11.11.566699; this version posted November 14, 2023. The copyright holder for this preprint (which was not certified by peer review) is the author/funder. This article is a US Government work. It is not subject to copyright under 17 USC 105 and is also made available for use under a CC0 license.

Figure S16. The binding site distance-difference category averages of the comparison between the active D3R and D2R structures.

The heatmap shows the difference of pairwise distance among the binding site residues between the D3R and D2R using their active structures (D3R – D2R) and categorized based on various $DD_{threshold}$ (T). Green represents the D3R conformation have a larger distance than D2R conformation, and brown represents the D2R conformation have a larger distance than D3R conformation. If the category average of the difference is smaller or equal to 0.9, it is colored as white in the masked heatmaps.

bioRxiv preprint doi: https://doi.org/10.1101/2023.11.11.566699; this version posted November 14, 2023. The copyright holder for this preprint (which was not certified by peer review) is the author/funder. This article is a US Government work. It is not subject to copyright under 17 USC 105 and is also made available for use under a CC0 license.

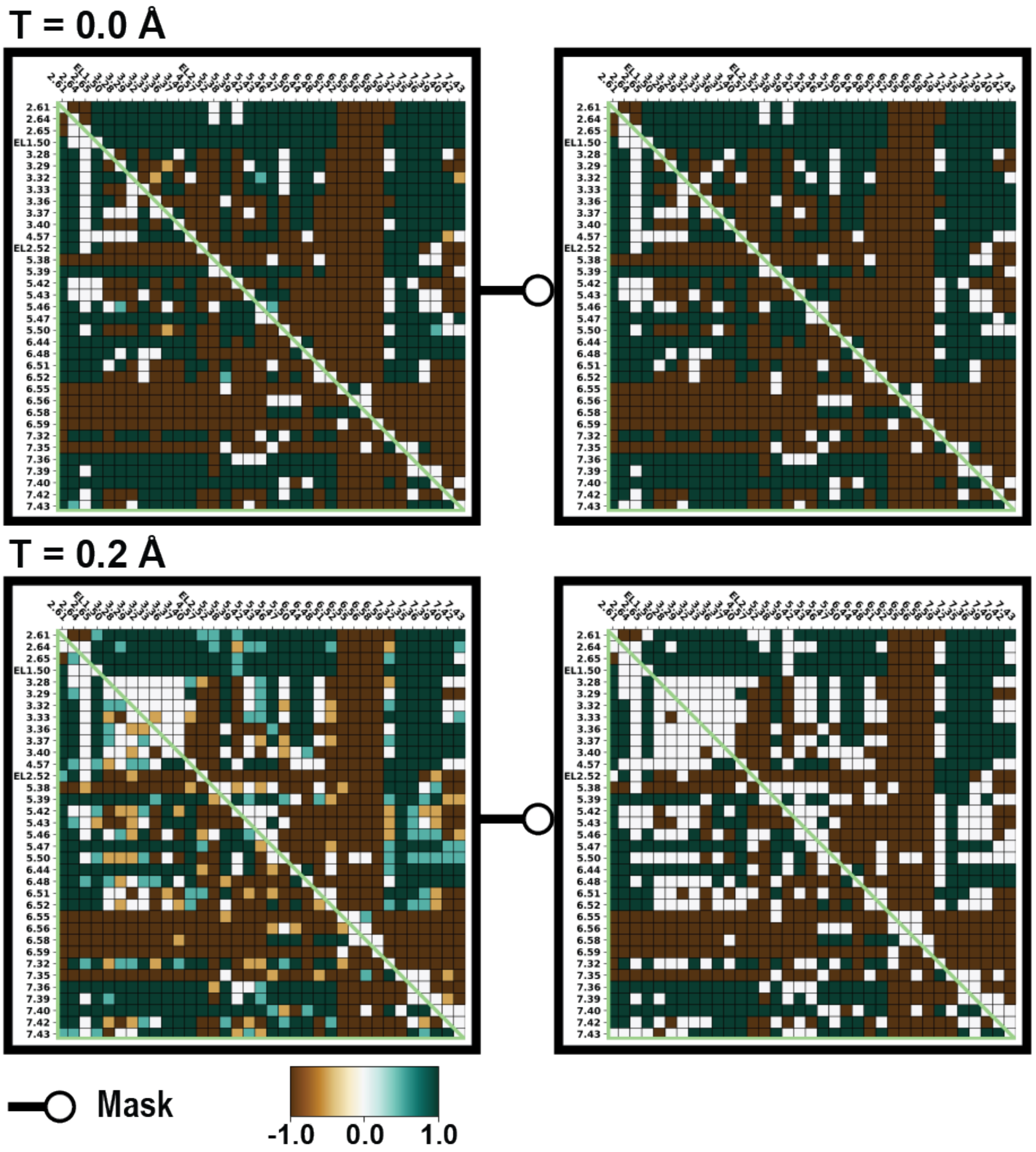

bioRxiv preprint doi: https://doi.org/10.1101/2023.11.11.566699; this version posted November 14, 2023. The copyright holder for this preprint (which was not certified by peer review) is the author/funder. This article is a US Government work. It is not subject to copyright under 17 USC 105 and is also made available for use under a CC0 license.

Figure S16. (continued)

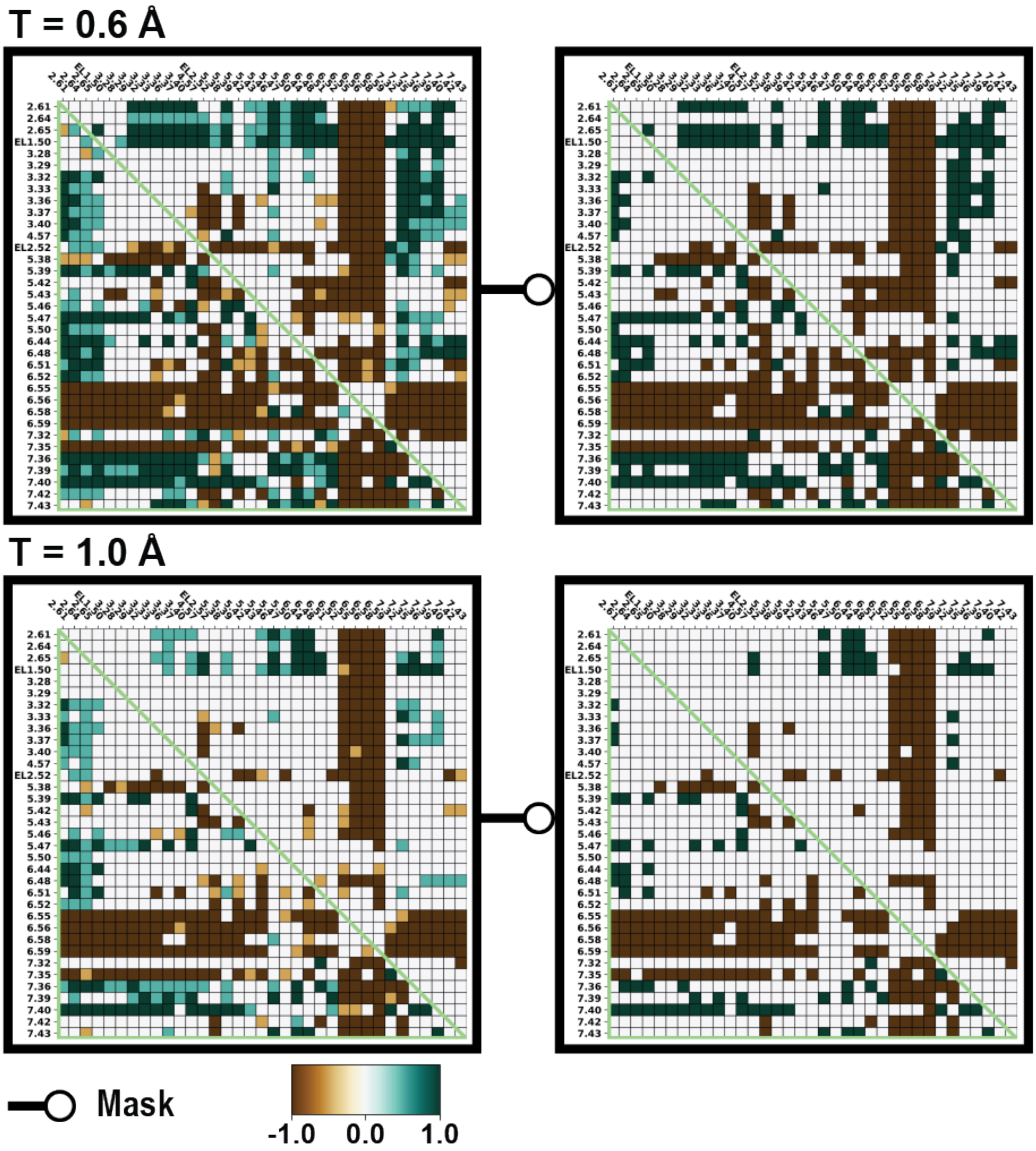

bioRxiv preprint doi: https://doi.org/10.1101/2023.11.11.566699; this version posted November 14, 2023. The copyright holder for this preprint (which was not certified by peer review) is the author/funder. This article is a US Government work. It is not subject to copyright under 17 USC 105 and is also made available for use under a CC0 license.

Figure S17. The OBS residue distance-difference category averages of the comparison between the active D3R and D2R structures.

The heatmap shows the difference of pairwise distance among the OBS residues between the D3R and D2R using their active structures (D3R – D2R) and categorized based on various $DD_{threshold}$ (T). Green represents the D3R conformation have a larger distance than D2R conformation, and brown represents the D2R conformation have a larger distance than D3R conformation. If the category average of the difference is smaller or equal to 0.9, it is colored as white in the masked heatmaps.

bioRxiv preprint doi: https://doi.org/10.1101/2023.11.11.566699; this version posted November 14, 2023. The copyright holder for this preprint (which was not certified by peer review) is the author/funder. This article is a US Government work. It is not subject to copyright under 17 USC 105 and is also made available for use under a CC0 license.

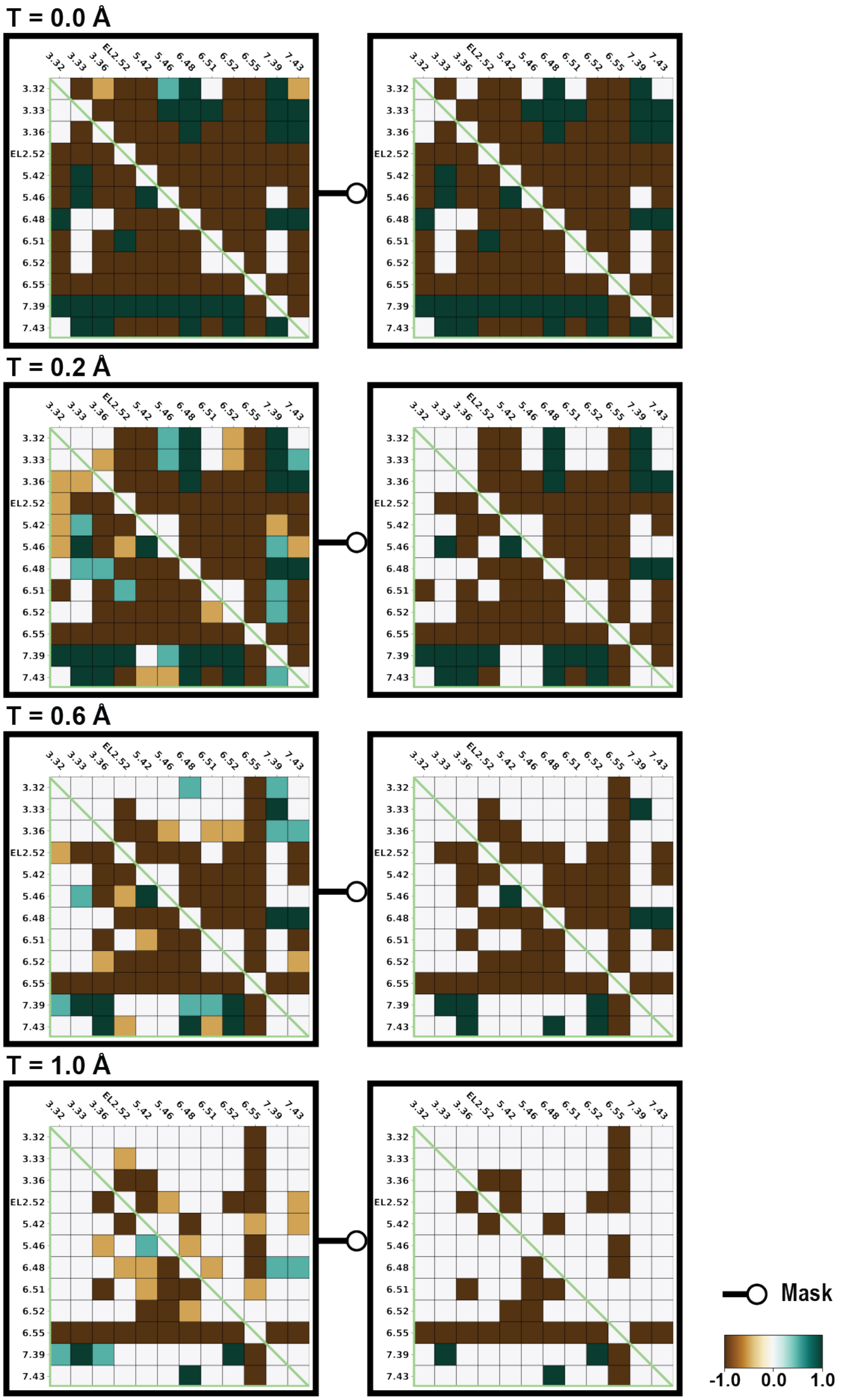

bioRxiv preprint doi: https://doi.org/10.1101/2023.11.11.566699; this version posted November 14, 2023. The copyright holder for this preprint (which was not certified by peer review) is the author/funder. This article is a US Government work. It is not subject to copyright under 17 USC 105 and is also made available for use under a CC0 license.

Figure S18. The subsegment distance-difference category averages of the comparison between the active D3R and D2R structures.

The heatmap shows the difference of pairwise distance among the subsegments between the D3R and D2R using their active structures (D3R – D2R) and categorized based on various $DD_{threshold}$ (T). Green represents the D3R conformation have a larger distance than D2R conformation, and brown represents the D2R conformation have a larger distance than D3R conformation. If the category average of the difference is smaller or equal to 0.9, it is colored as white in the masked heatmaps.

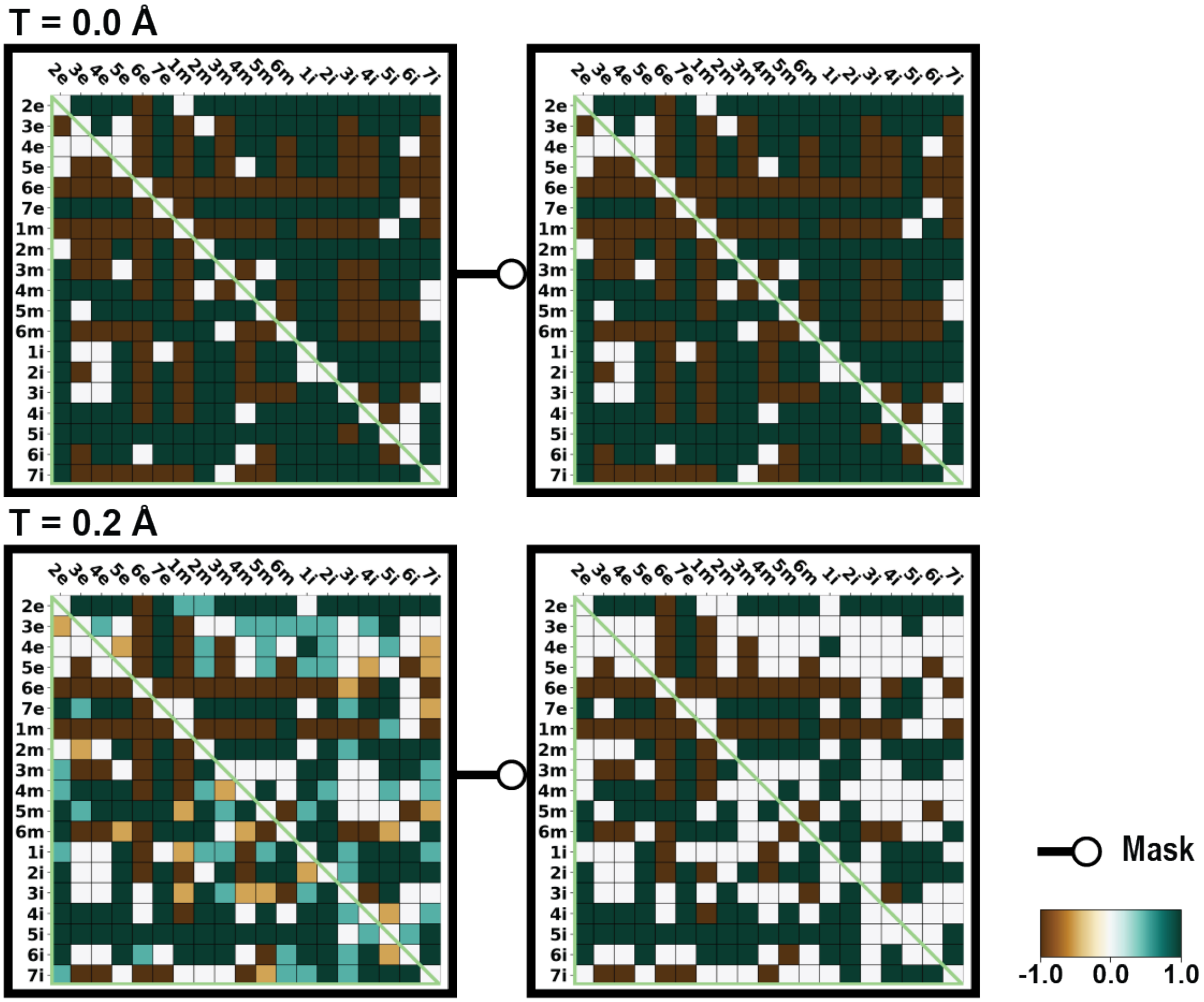

bioRxiv preprint doi: https://doi.org/10.1101/2023.11.11.566699; this version posted November 14, 2023. The copyright holder for this preprint (which was not certified by peer review) is the author/funder. This article is a US Government work. It is not subject to copyright under 17 USC 105 and is also made available for use under a CC0 license.

Figure S18. (continued)

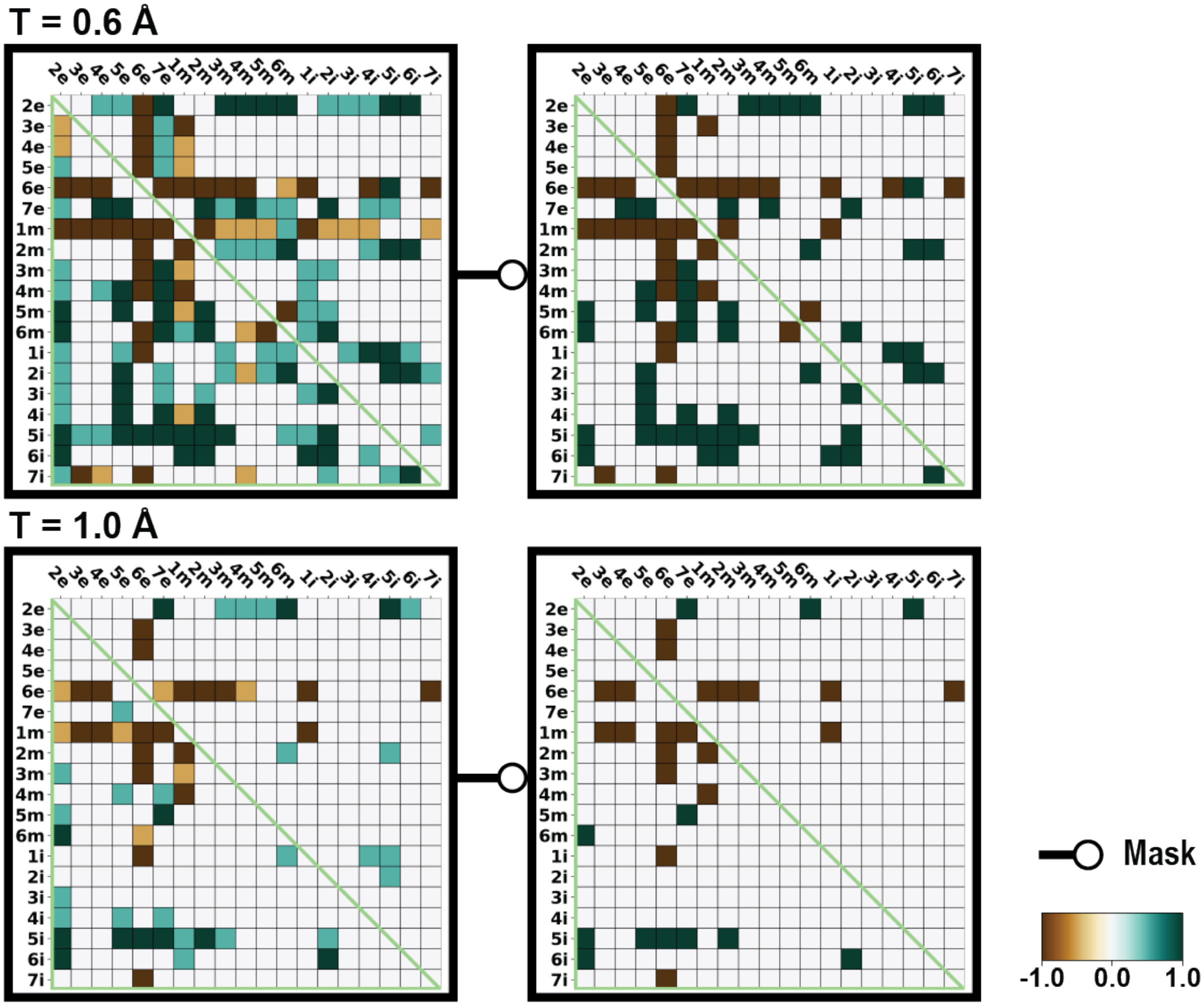

bioRxiv preprint doi: https://doi.org/10.1101/2023.11.11.566699; this version posted November 14, 2023. The copyright holder for this preprint (which was not certified by peer review) is the author/funder. This article is a US Government work. It is not subject to copyright under 17 USC 105 and is also made available for use under a CC0 license.

Figure S19. The binding site distance-difference category averages of both the comparisons between the active and between the inactive D3R and D2R structures.

The heatmap was combining the derived categories from inactive pairs (**Fig. S13**) and active pairs (**Fig. S16**) with various $DD_{threshold}$ (T). This heatmap revealed common patterns in distance changes (D3R – D2R) for both inactive and active states of these two receptors. The color green signifies a more distance change in D3R, while brown indicates a greater change in D2R. In instances where the category average of the difference is 0.9 or less, the corresponding region in the masked heatmaps is represented in white.

bioRxiv preprint doi: https://doi.org/10.1101/2023.11.11.566699; this version posted November 14, 2023. The copyright holder for this preprint (which was not certified by peer review) is the author/funder. This article is a US Government work. It is not subject to copyright under 17 USC 105 and is also made available for use under a CC0 license.

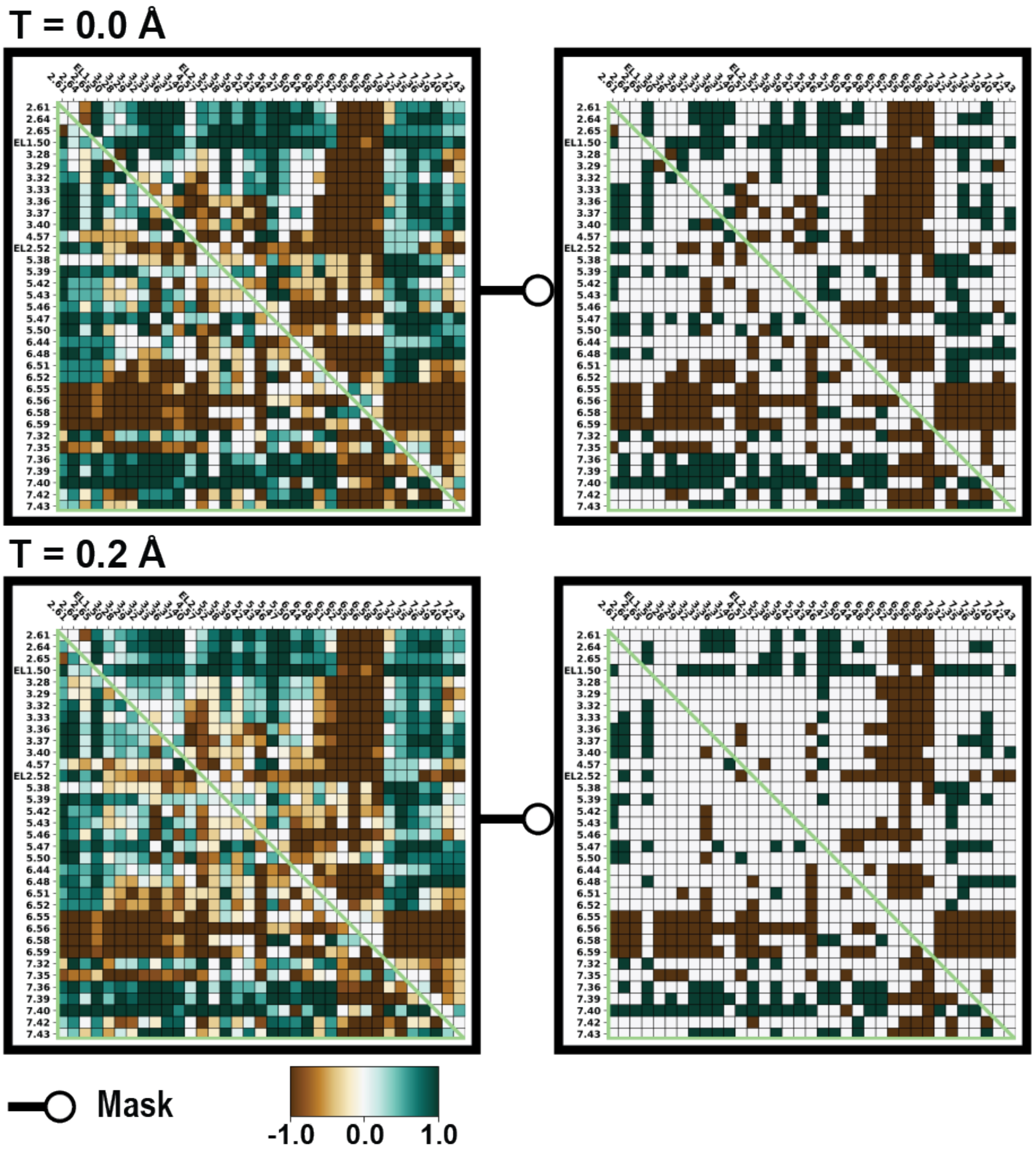

bioRxiv preprint doi: https://doi.org/10.1101/2023.11.11.566699; this version posted November 14, 2023. The copyright holder for this preprint (which was not certified by peer review) is the author/funder. This article is a US Government work. It is not subject to copyright under 17 USC 105 and is also made available for use under a CC0 license.

Figure S19. (continued)

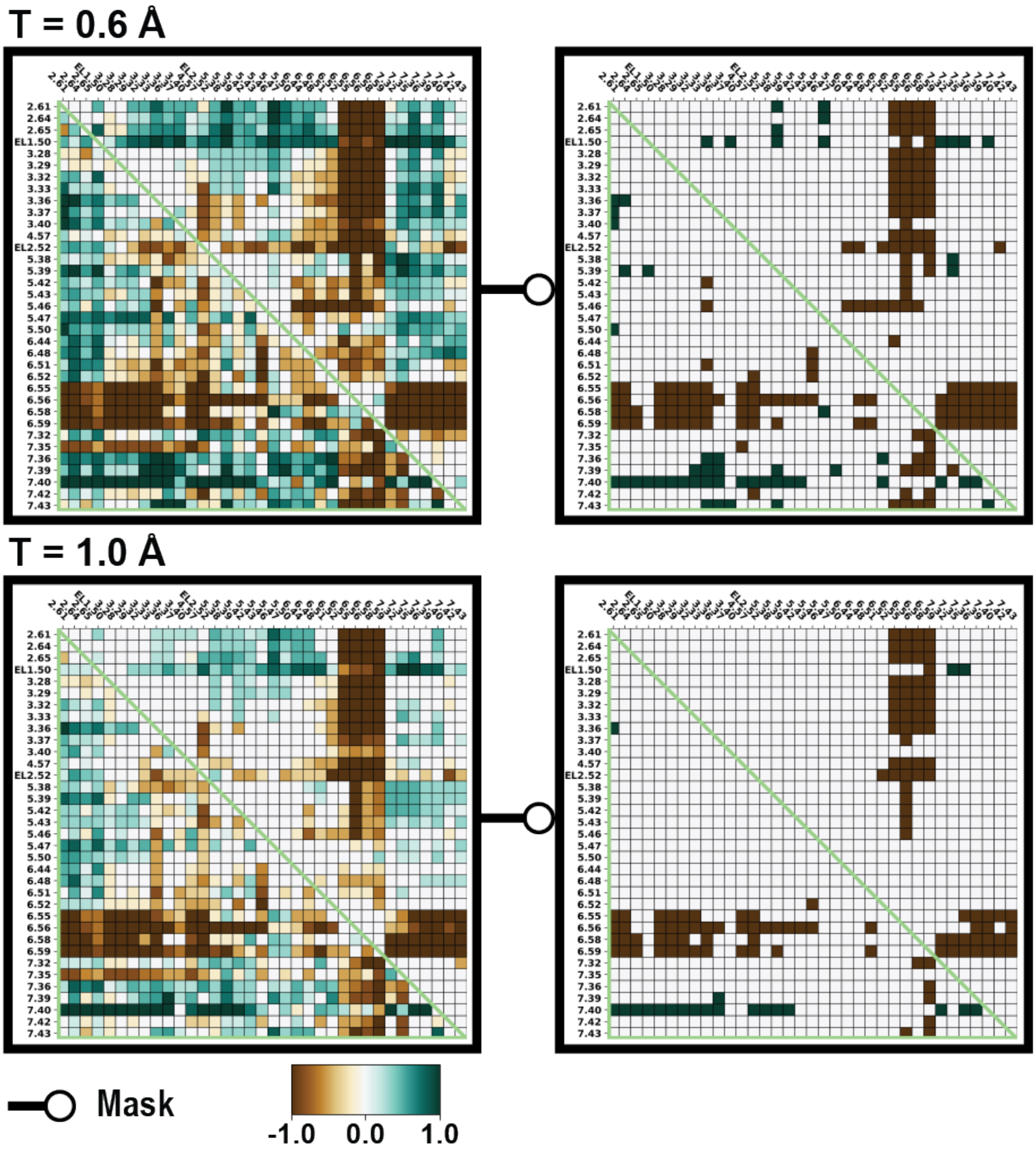

bioRxiv preprint doi: https://doi.org/10.1101/2023.11.11.566699; this version posted November 14, 2023. The copyright holder for this preprint (which was not certified by peer review) is the author/funder. This article is a US Government work. It is not subject to copyright under 17 USC 105 and is also made available for use under a CC0 license.

Figure S20. The OBS residue distance-difference category averages of both the comparisons between the active and between the inactive D3R and D2R structures.

The heatmap was combining the derived categories from inactive pairs (**Fig. S14**) and active pairs (**Fig. S17**) with various $DD_{threshold}$ (T). This heatmap revealed common patterns in distance changes (D3R – D2R) for both inactive and active states of these two receptors. The color green signifies a more distance change in D3R, while brown indicates a greater change in D2R. In instances where the category average of the difference is 0.9 or less, the corresponding region in the masked heatmaps is represented in white.

bioRxiv preprint doi: https://doi.org/10.1101/2023.11.11.566699; this version posted November 14, 2023. The copyright holder for this preprint (which was not certified by peer review) is the author/funder. This article is a US Government work. It is not subject to copyright under 17 USC 105 and is also made available for use under a CC0 license.

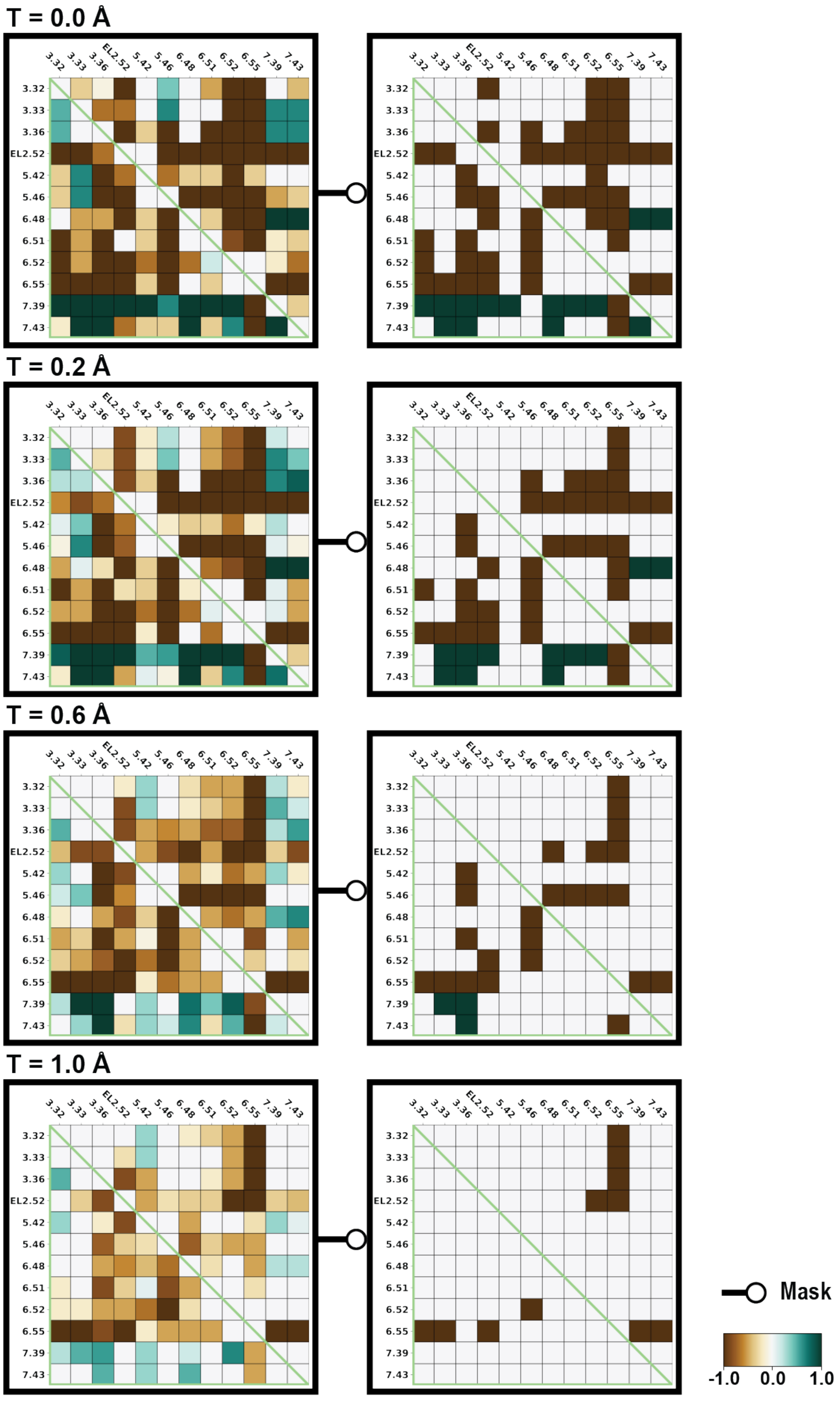

bioRxiv preprint doi: https://doi.org/10.1101/2023.11.11.566699; this version posted November 14, 2023. The copyright holder for this preprint (which was not certified by peer review) is the author/funder. This article is a US Government work. It is not subject to copyright under 17 USC 105 and is also made available for use under a CC0 license.

Figure S21. The subsegment distance-difference category averages of both the comparisons between the active and between the inactive D3R and D2R structures.

The heatmap was combining the derived categories from inactive pairs (**Fig. S15**) and active pairs (**Fig. S18**) with various $DD_{threshold}$ (T). This heatmap revealed common patterns in distance changes (D3R – D2R) for both inactive and active states of these two receptors. The color green signifies a more distance change in D3R, while brown indicates a greater change in D2R. In instances where the category average of the difference is 0.9 or less, the corresponding region in the masked heatmaps is represented in white.

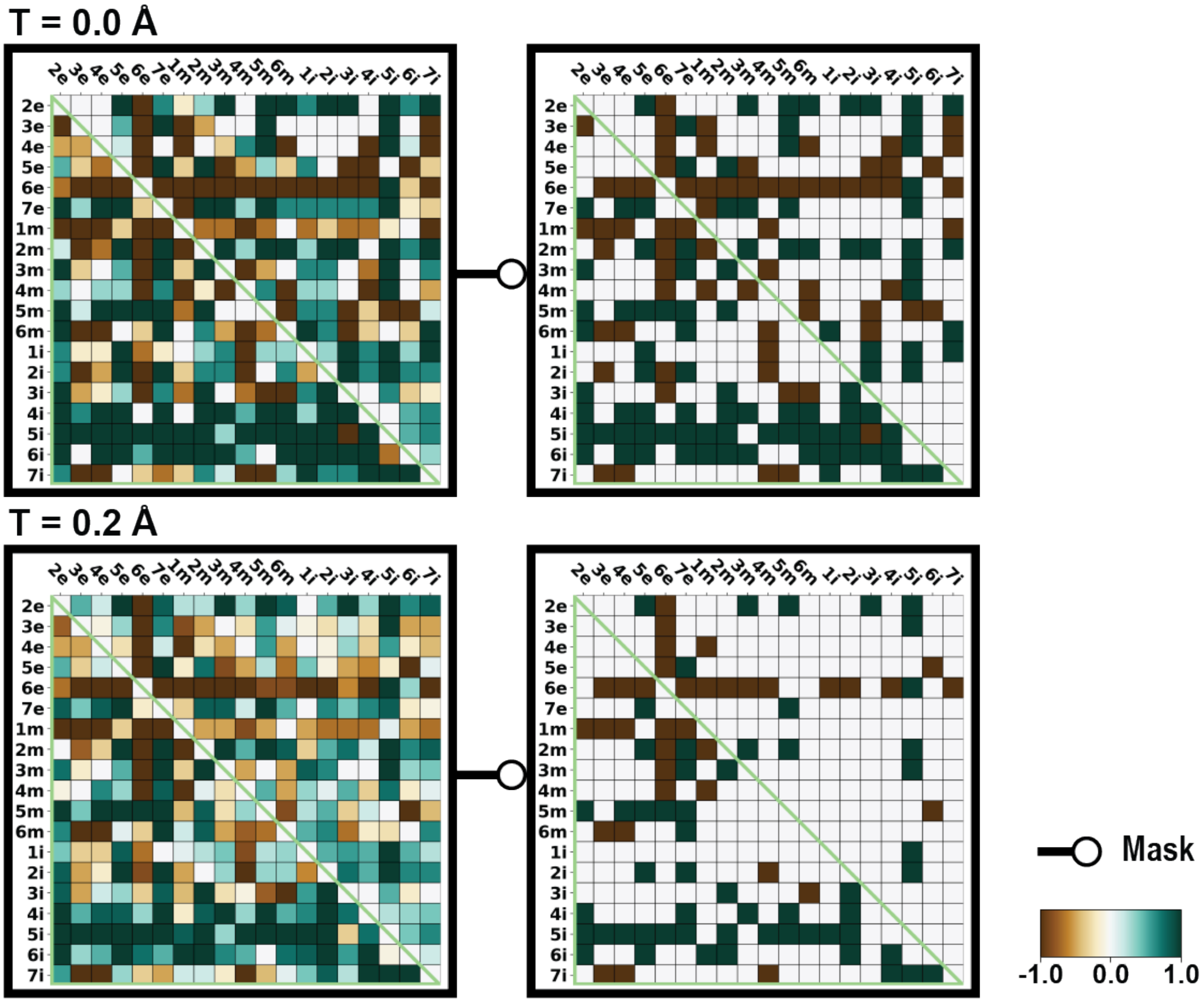

bioRxiv preprint doi: https://doi.org/10.1101/2023.11.11.566699; this version posted November 14, 2023. The copyright holder for this preprint (which was not certified by peer review) is the author/funder. This article is a US Government work. It is not subject to copyright under 17 USC 105 and is also made available for use under a CC0 license.

Figure S21. (continued)

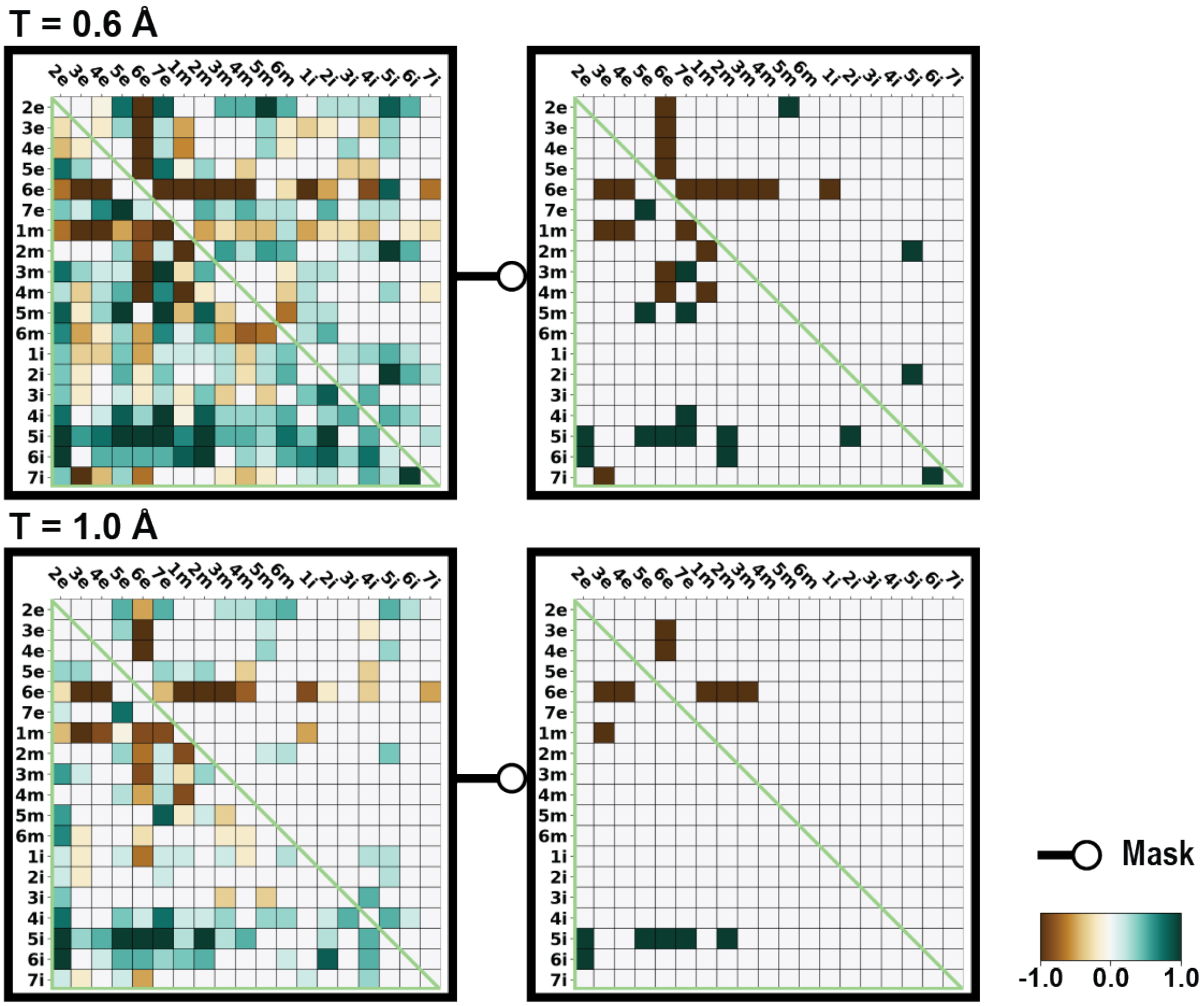

bioRxiv preprint doi: https://doi.org/10.1101/2023.11.11.566699; this version posted November 14, 2023. The copyright holder for this preprint (which was not certified by peer review) is the author/funder. This article is a US Government work. It is not subject to copyright under 17 USC 105 and is also made available for use under a CC0 license.

Figure S22. Proline kinks and the ligand binding residues dynamics in the MD simulations

This proline kink (Prokink) measurement was conducted for TM5, TM6, and TM7 using the indicated MD trajectories. Note that the control MD simulations refers to those of D3R/G_i-PD128907, D3R/G_i-pramipexole, and D2R/G_i-bromocriptine. Panel A, B, and C shows the Prokink of TM5, TM6 and TM7 of control simulations, respectively. In panel D, E, and F, the Prokink of TM5, TM6 and TM7 of quinpirole simulations were shown. We assessed the Prokink bend angle with Simulaid. In the calculations, seven residues preceding and succeeding proline were employed for TM5 and TM6, while for TM7, the calculations utilized five residues before and after the proline. The pairwise RMSD of backbone of ligand binding residues were calculated for the same bootstrapped ensemble for control simulations (G) and quinpirole simulations (H). For D3R/Gi-PD128907, D3R/Gi-pramipexole, and D2R/Gi-bromocriptine, the peak positions are 0.97 Å, 0.80 Å, and 0.76 Å, respectively. The peak positions for D2R/Gi-bromocriptine and D3R/Gi-5R-quinpirole are 0.97 Å and 0.77 Å, respectively.

bioRxiv preprint doi: https://doi.org/10.1101/2023.11.11.566699; this version posted November 14, 2023. The copyright holder for this preprint (which was not certified by peer review) is the author/funder. This article is a US Government work. It is not subject to copyright under 17 USC 105 and is also made available for use under a CC0 license.

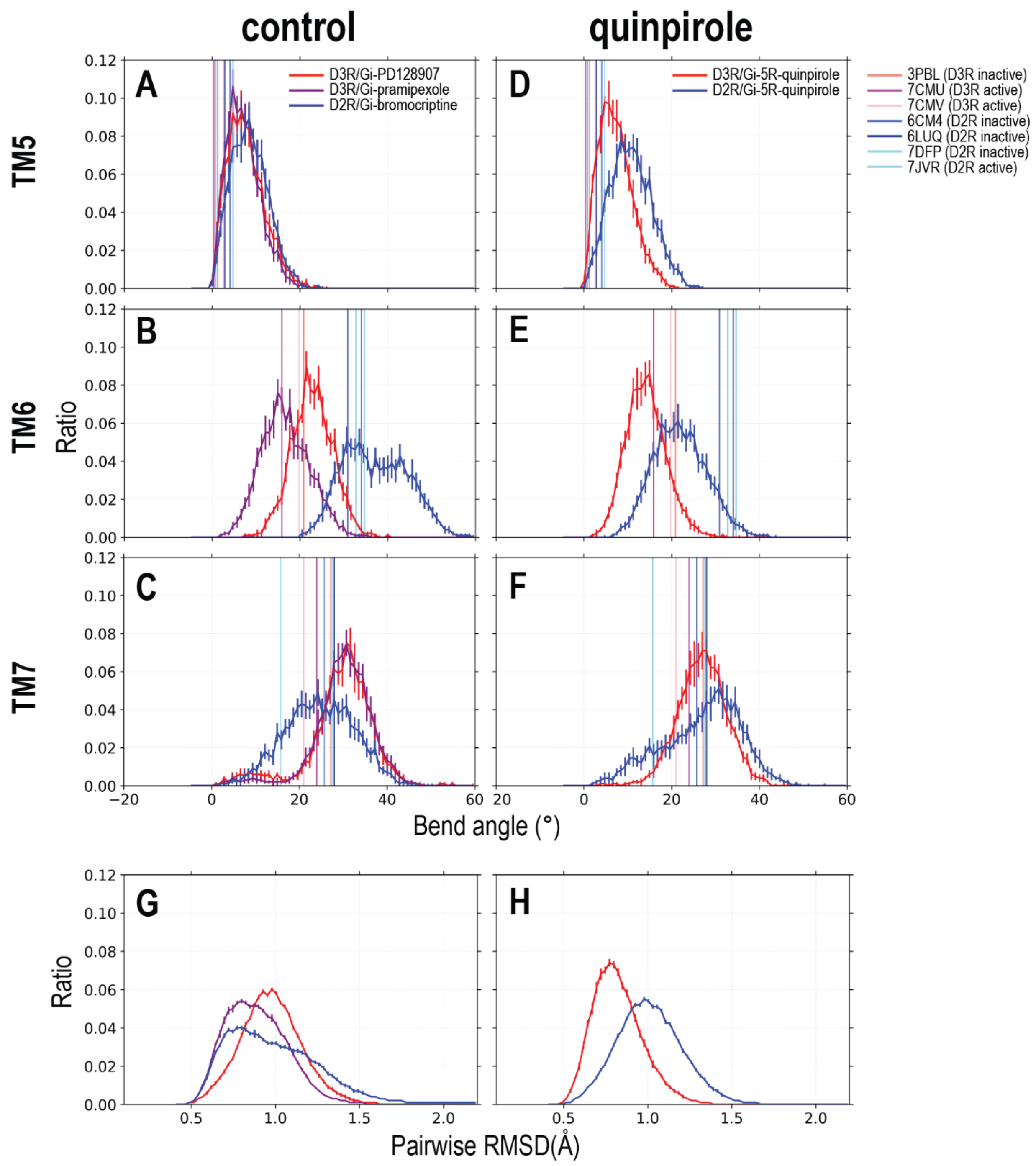

bioRxiv preprint doi: https://doi.org/10.1101/2023.11.11.566699; this version posted November 14, 2023. The copyright holder for this preprint (which was not certified by peer review) is the author/funder. This article is a US Government work. It is not subject to copyright under 17 USC 105 and is also made available for use under a CC0 license.

Figure S23. Quinpirole prefers to be in the 5R-isomer in the ligand binding site of D2R.

In panel A, the evolutions of the z positions (i.e., along the axis perpendicular to the membrane) of the ligands in both 5S-isomer and 5R-isomer are shown, while the combined distributions of the z positions for these two isomers are shown on the right. When the binding pose of the 5S-isomer was relatively stable, it still exhibited higher z position values compared to the 5R-isomer. The averaged pairwise ligand RMSDs for each MD trajectory are presented in panel B, indicating that the 5R-isomer was more stable than the 5S-isomer. In panel C, the results of the MMGB/SA calculations using the stable trajectories of the 5S- and 5R-isomers are shown, demonstrating the 5R-isomer was bound tighter than the 5S-isomer.

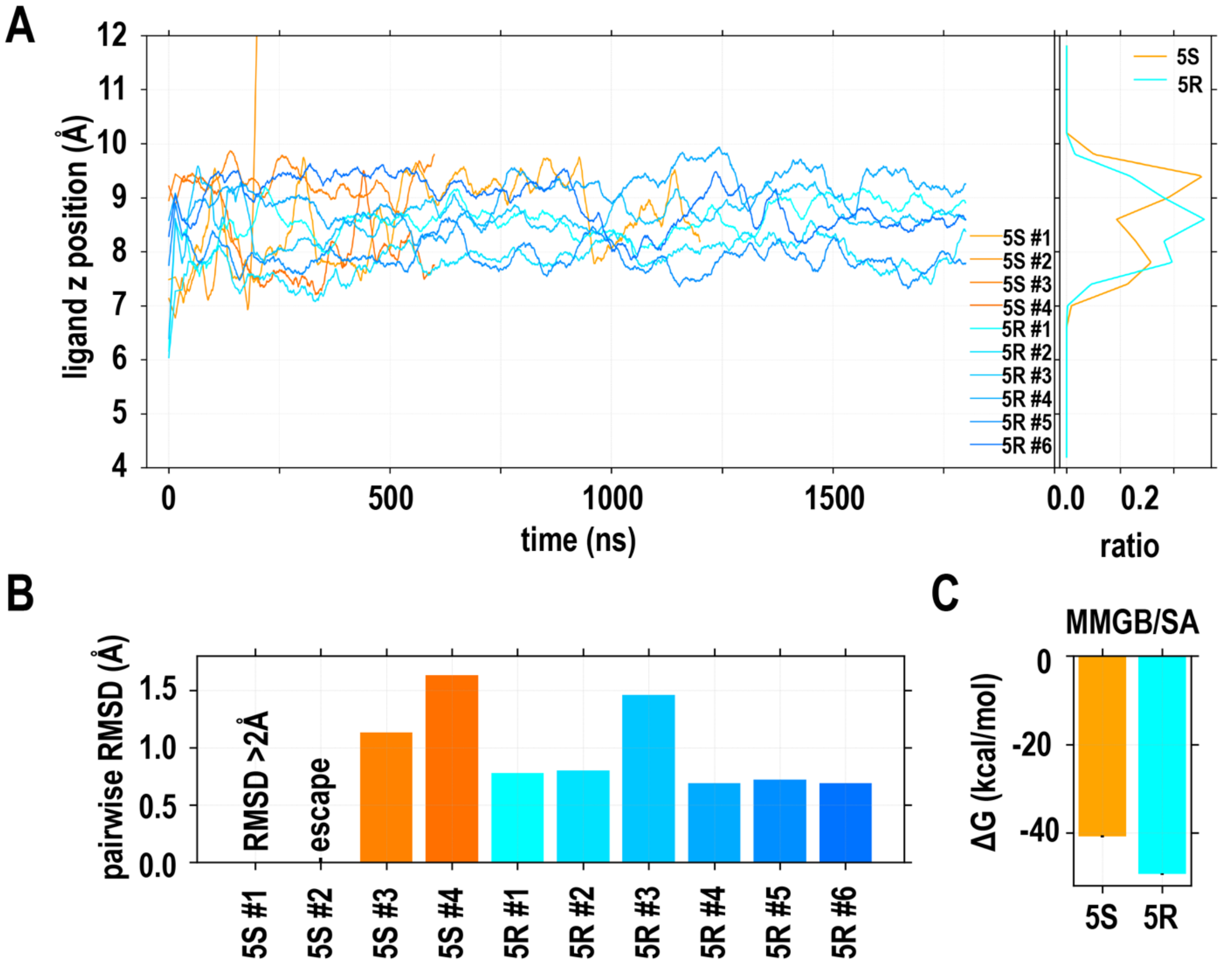

bioRxiv preprint doi: https://doi.org/10.1101/2023.11.11.566699; this version posted November 14, 2023. The copyright holder for this preprint (which was not certified by peer review) is the author/funder. This article is a US Government work. It is not subject to copyright under 17 USC 105 and is also made available for use under a CC0 license.

Figure S24. Trp$^{7.40}$ χ1 angle distribution

The χ1 angle distribution was analyzed for two sets of the indicated simulations. For each set of simulations, the χ1 angle distribution was calculated for residue Trp$^{7.40}$. In the plots, the vertical lines indicate the experimentally determined D2R and D3R structures, which serve as references for comparison of the distributions obtained from the simulations.

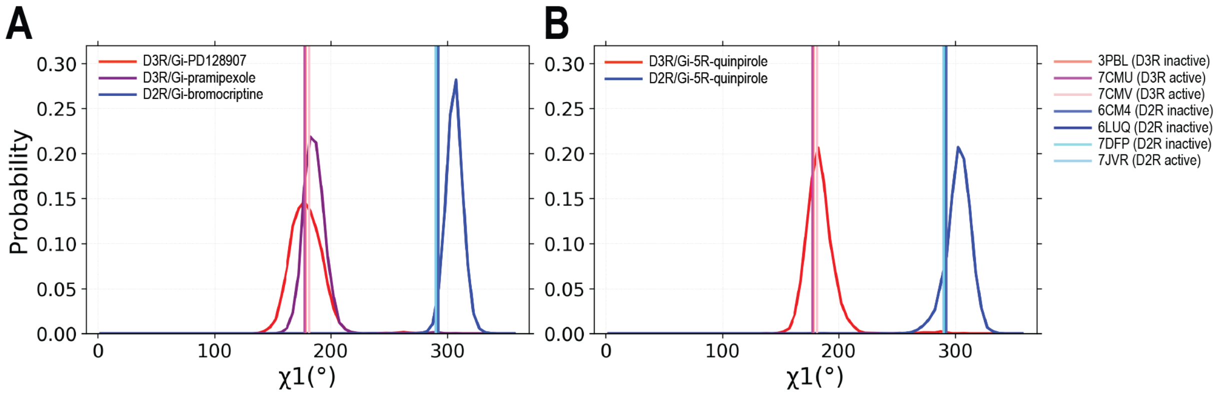

bioRxiv preprint doi: https://doi.org/10.1101/2023.11.11.566699; this version posted November 14, 2023. The copyright holder for this preprint (which was not certified by peer review) is the author/funder. This article is a US Government work. It is not subject to copyright under 17 USC 105 and is also made available for use under a CC0 license.

Figure S25. Different conformations of Trp$^{7.40}$ in the 5R-quinpirole bound D3R and D2R.

To investigate interactions between Trp$^{7.40}$ and Ser$^{1.38}$ in D3R or Leu$^{1.38}$ in D2R, the closest heavy atom contacting distance between residues 7.40 and 1.38 was calculated. The results show that in the D3R, there is a tendency to form a water-mediated hydrogen bond between these two residues, whereas in the D2R, such an interaction is not observed. (A) The reaction coordinate is defined by the distance of Trp$^{7.40}$– Glu$^{2.65}$ and Trp$^{7.40}$– Ser$^{1.38}$ for D3R, and Trp$^{7.40}$–Leu$^{1.38}$ for the D2R. (B) The reaction coordinate is determined using the χ1 angles of Trp$^{7.40}$ and Thr$^{7.38}$ for the D3R, and Trp$^{7.40}$ and Phe$^{7.38}$ for the D2R. The various symbols indicate the corresponding values in the experimentally determined structures.

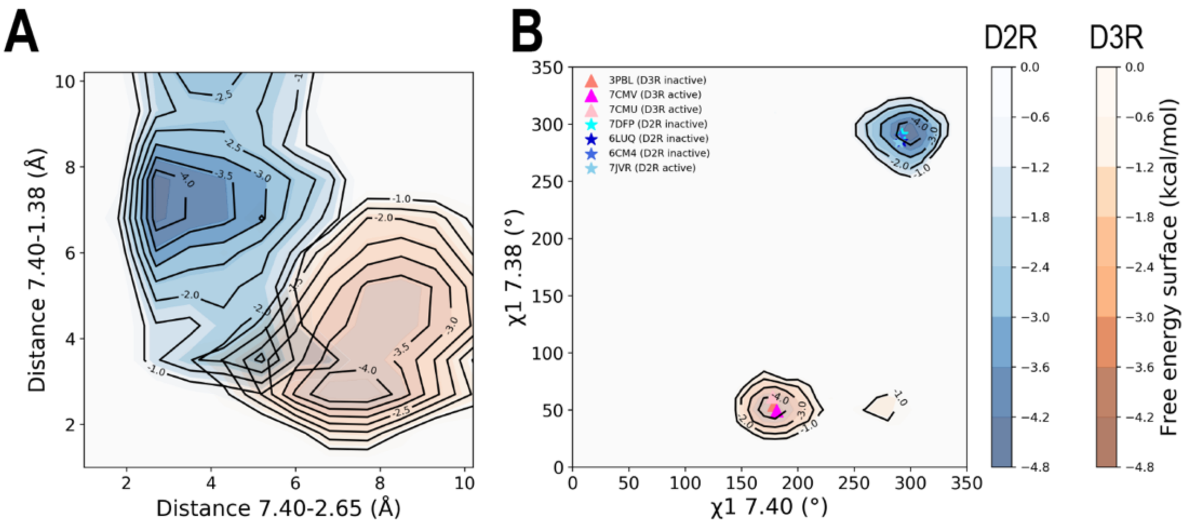

bioRxiv preprint doi: https://doi.org/10.1101/2023.11.11.566699; this version posted November 14, 2023. The copyright holder for this preprint (which was not certified by peer review) is the author/funder. This article is a US Government work. It is not subject to copyright under 17 USC 105 and is also made available for use under a CC0 license.

Figure S26. The binding site distance-difference category averages of the comparisons between the D3R and D2R MD simulations.

The heatmap shows the difference of pairwise distance among the binding site residues between the D3R and D2R using their active structures (D3R – D2R) and categorized based on various $DD_{threshold}$ (T). Green represents the D3R conformation have a larger distance than D2R conformation, and brown represents the D2R conformation have a larger distance than D3R conformation. Note that the MD simulations from D3R/G_i-PD128907, D3R/G_i-pramipexole, and D2R/G_i-bromocriptine are referred as “control”, and simulations from D3R/G_i-5R-quinpirole, and D2R/G_i-5R-quinpirole are referred as “quinpirole”.

bioRxiv preprint doi: https://doi.org/10.1101/2023.11.11.566699; this version posted November 14, 2023. The copyright holder for this preprint (which was not certified by peer review) is the author/funder. This article is a US Government work. It is not subject to copyright under 17 USC 105 and is also made available for use under a CC0 license.

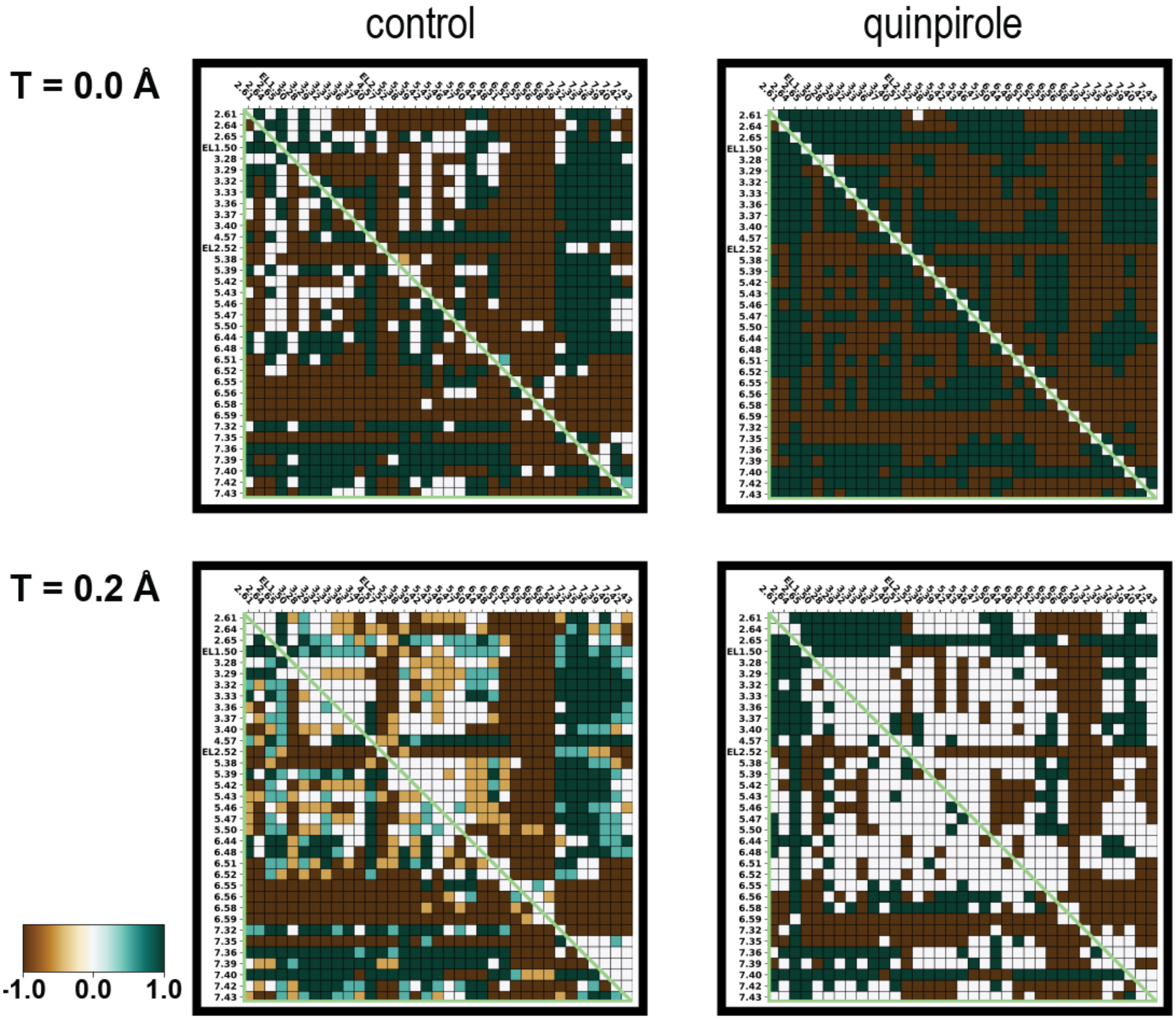

bioRxiv preprint doi: https://doi.org/10.1101/2023.11.11.566699; this version posted November 14, 2023. The copyright holder for this preprint (which was not certified by peer review) is the author/funder. This article is a US Government work. It is not subject to copyright under 17 USC 105 and is also made available for use under a CC0 license.

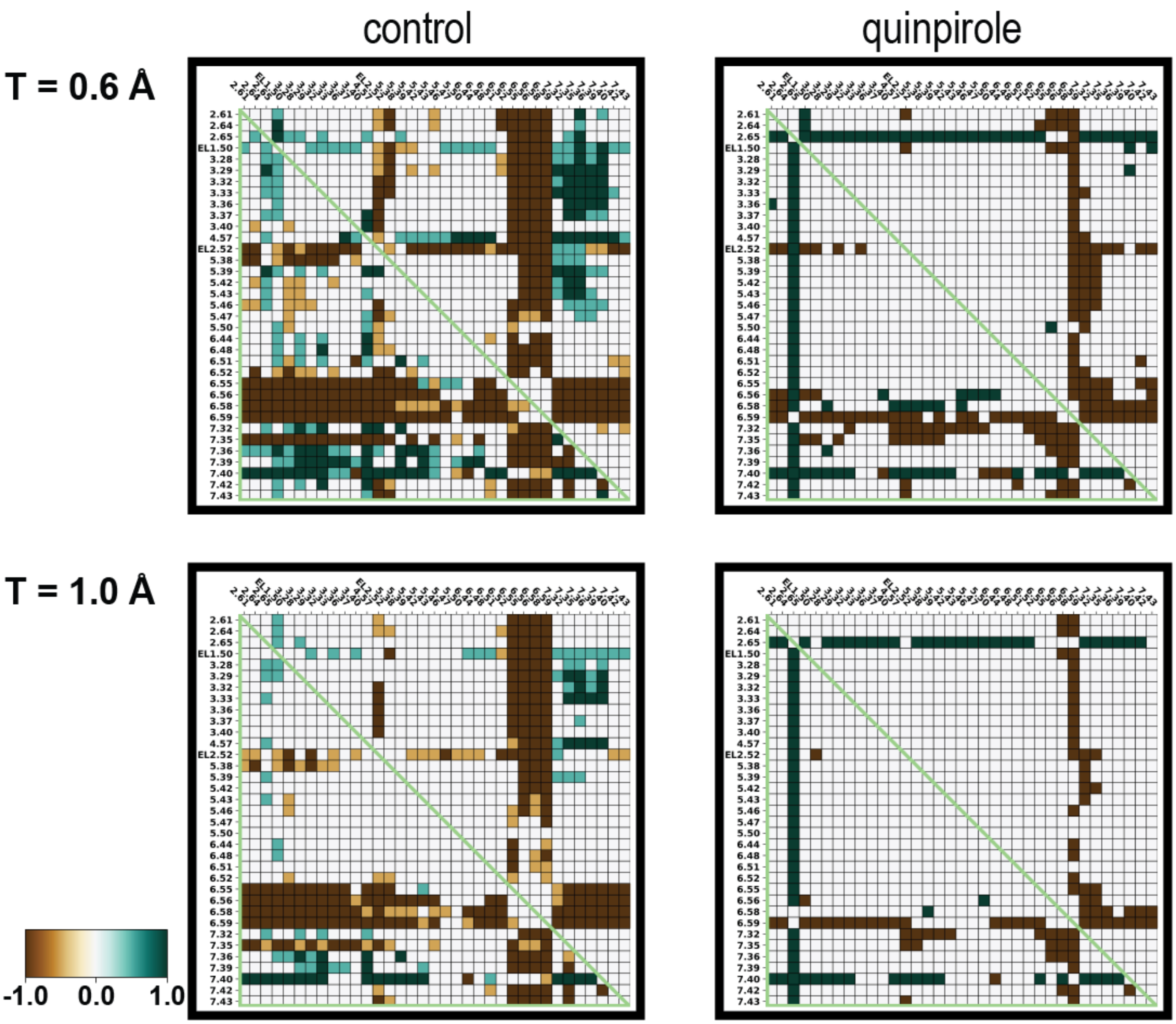

bioRxiv preprint doi: https://doi.org/10.1101/2023.11.11.566699; this version posted November 14, 2023. The copyright holder for this preprint (which was not certified by peer review) is the author/funder. This article is a US Government work. It is not subject to copyright under 17 USC 105 and is also made available for use under a CC0 license.

Figure S27. The OBS residue distance-difference category averages of the comparisons between the D3R and D2R MD simulations.

The heatmap shows the difference of pairwise distance among the binding site residues between the D3R and D2R using their active structures (D3R – D2R) and categorized based on various $DD_{threshold}$ (T). Green represents the D3R conformation have a larger distance than D2R conformation, and brown represents the D2R conformation have a larger distance than D3R conformation. Note that the MD simulations from D3R/G_i-PD128907, D3R/G_i-pramipexole, and D2R/G_i-bromocriptine are referred as “control”, and simulations from D3R/G_i-5R-quinpirole, and D2R/G_i-5R-quinpirole are referred as “quinpirole”.

bioRxiv preprint doi: https://doi.org/10.1101/2023.11.11.566699; this version posted November 14, 2023. The copyright holder for this preprint (which was not certified by peer review) is the author/funder. This article is a US Government work. It is not subject to copyright under 17 USC 105 and is also made available for use under a CC0 license.

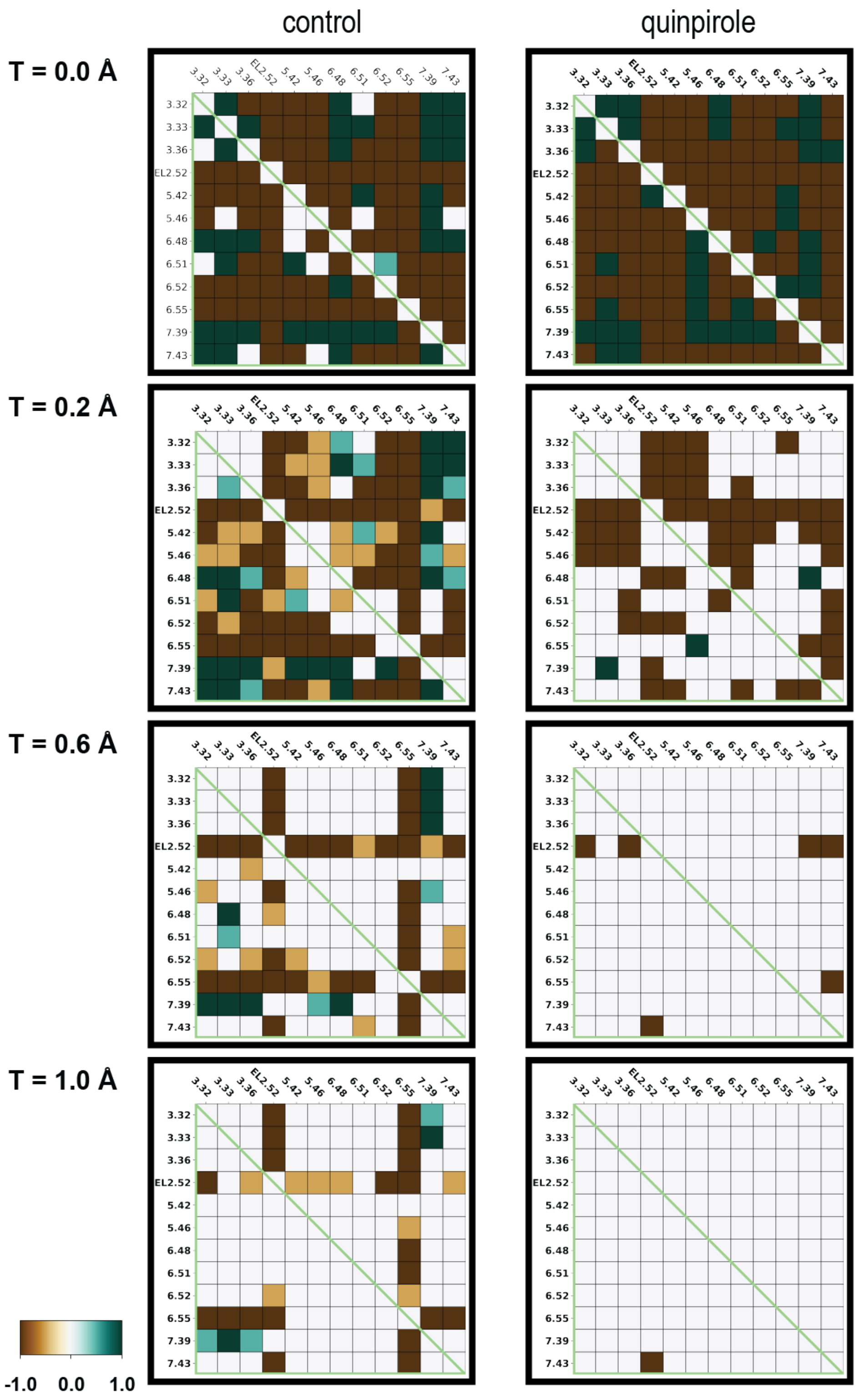

bioRxiv preprint doi: https://doi.org/10.1101/2023.11.11.566699; this version posted November 14, 2023. The copyright holder for this preprint (which was not certified by peer review) is the author/funder. This article is a US Government work. It is not subject to copyright under 17 USC 105 and is also made available for use under a CC0 license.

Figure S28. The subsegment distance-difference category averages of the comparisons between the D3R and D2R MD simulations.

The heatmap shows the difference of pairwise distance among the binding site residues between the D3R and D2R using their active structures (D3R – D2R) and categorized based on various $DD_{threshold}$ (T). Green represents the D3R conformation have a larger distance than D2R conformation, and brown represents the D2R conformation have a larger distance than D3R conformation. Note that the MD simulations from D3R/G_i-PD128907, D3R/G_i-pramipexole, and D2R/G_i-bromocriptine are referred as "control", and simulations from D3R/G_i-5R-quinpirole, and D2R/G_i-5R-quinpirole are referred as "quinpirole".

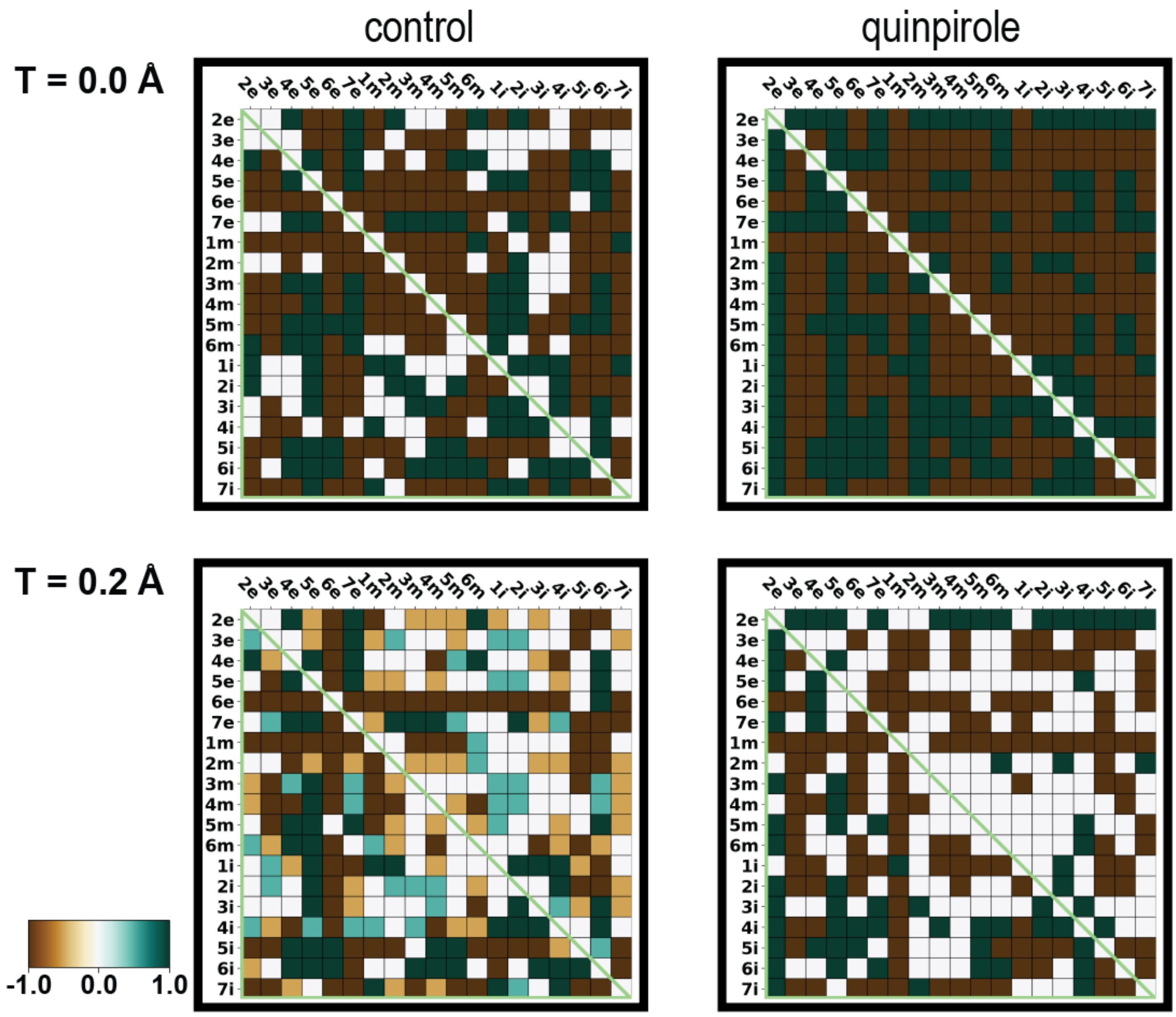

bioRxiv preprint doi: https://doi.org/10.1101/2023.11.11.566699; this version posted November 14, 2023. The copyright holder for this preprint (which was not certified by peer review) is the author/funder. This article is a US Government work. It is not subject to copyright under 17 USC 105 and is also made available for use under a CC0 license.

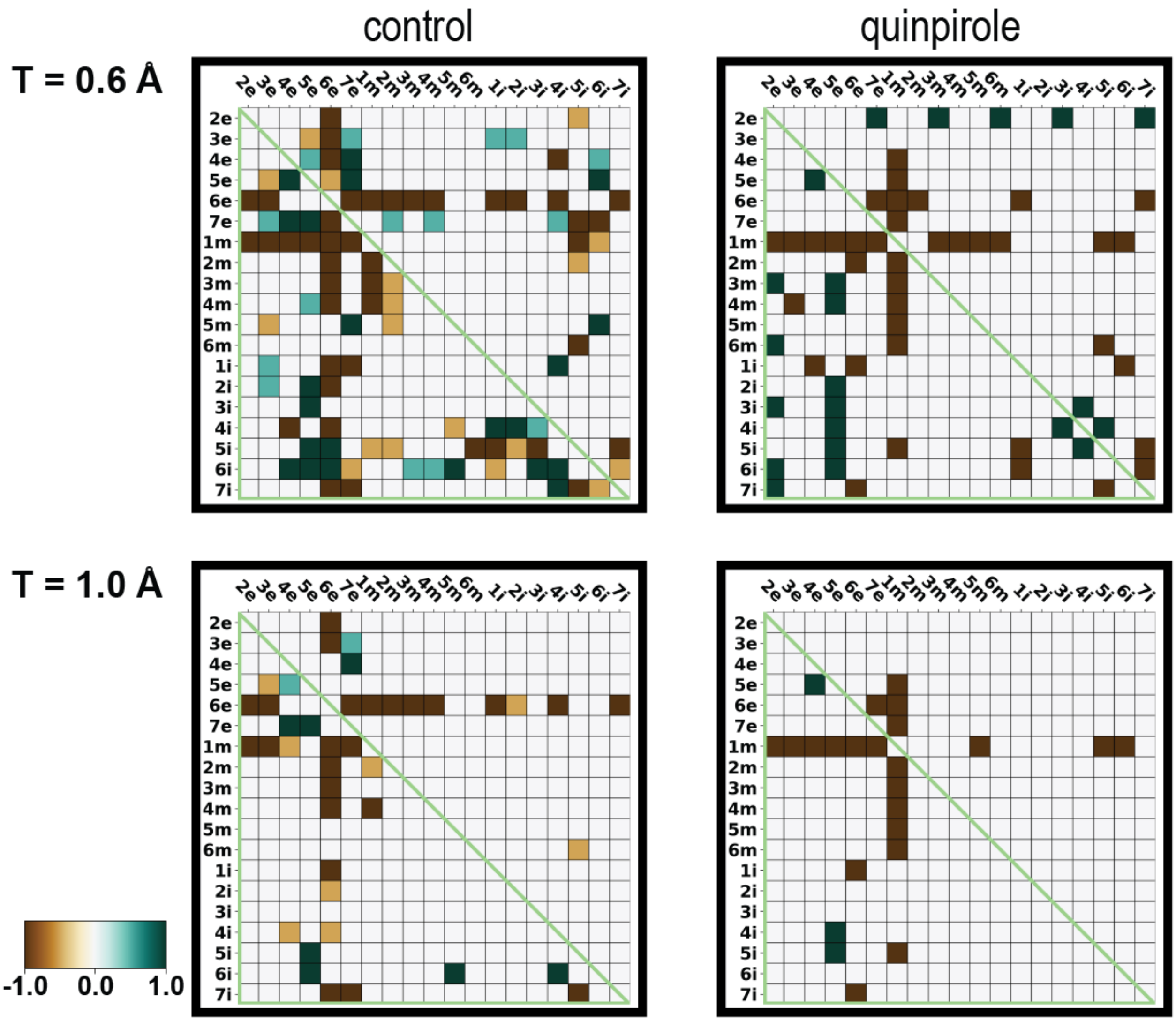

bioRxiv preprint doi: https://doi.org/10.1101/2023.11.11.566699; this version posted November 14, 2023. The copyright holder for this preprint (which was not certified by peer review) is the author/funder. This article is a US Government work. It is not subject to copyright under 17 USC 105 and is also made available for use under a CC0 license.

References

(1) Im, D.; Inoue, A.; Fujiwara, T.; Nakane, T.; Yamanaka, Y.; Uemura, T.; Mori, C.; Shiimura, Y.; Kimura, K. T.; Asada, H.; Nomura, N.; Tanaka, T.; Yamashita, A.; Nango, E.; Tono, K.; Kadji, F. M. N.; Aoki, J.; Iwata, S.; Shimamura, T., Structure of the Dopamine D2 Receptor in Complex with the Antipsychotic Drug Spiperone. *Nat Commun* **2020**, 11, 6442.

(2) Fan, L.; Tan, L.; Chen, Z.; Qi, J.; Nie, F.; Luo, Z.; Cheng, J.; Wang, S., Haloperidol Bound D2 Dopamine Receptor Structure Inspired the Discovery of Subtype Selective Ligands. *Nat Commun* **2020**, 11, 1074.

(3) Wang, S.; Che, T.; Levit, A.; Shoichet, B. K.; Wacker, D.; Roth, B. L., Structure of the D2 Dopamine Receptor Bound to the Atypical Antipsychotic Drug Risperidone. *Nature* **2018**, 555, 269-273.

(4) Zhuang, Y.; Xu, P.; Mao, C.; Wang, L.; Krumm, B.; Zhou, X. E.; Huang, S.; Liu, H.; Cheng, X.; Huang, X. P.; Shen, D. D.; Xu, T.; Liu, Y. F.; Wang, Y.; Guo, J.; Jiang, Y.; Jiang, H.; Melcher, K.; Roth, B. L.; Zhang, Y.; Zhang, C.; Xu, H. E., Structural Insights into the Human D1 and D2 Dopamine Receptor Signaling Complexes. *Cell* **2021**, 184, 931-942 e18.

(5) Chien, E. Y.; Liu, W.; Zhao, Q.; Katritch, V.; Han, G. W.; Hanson, M. A.; Shi, L.; Newman, A. H.; Javitch, J. A.; Cherezov, V.; Stevens, R. C., Structure of the Human Dopamine D3 Receptor in Complex with a D2/D3 Selective Antagonist. *Science* **2010**, 330, 1091-5.

(6) Xu, P.; Huang, S.; Mao, C.; Krumm, B. E.; Zhou, X. E.; Tan, Y.; Huang, X. P.; Liu, Y.; Shen, D. D.; Jiang, Y.; Yu, X.; Jiang, H.; Melcher, K.; Roth, B. L.; Cheng, X.; Zhang, Y.; Xu, H. E., Structures of the Human Dopamine D3 Receptor-Gi Complexes. *Mol Cell* **2021**, 81, 1147-1159 e4.

(7) Yin, J.; Chen, K. M.; Clark, M. J.; Hijazi, M.; Kumari, P.; Bai, X. C.; Sunahara, R. K.; Barth, P.; Rosenbaum, D. M., Structure of a D2 Dopamine Receptor-G-Protein Complex in a Lipid Membrane. *Nature* **2020**, 584, 125-129.

bioRxiv preprint doi: https://doi.org/10.1101/2023.11.11.566699; this version posted November 14, 2023. The copyright holder for this preprint (which was not certified by peer review) is the author/funder. This article is a US Government work. It is not subject to copyright under 17 USC 105 and is also made available for use under a CC0 license.

(8) Mezei, M., Simulaid: A Simulation Facilitator and Analysis Program. *J Comput Chem* **2010**, 31, 2658-68.
